# Pillared Mo_2_TiC_2_ MXene for high-power and long-life lithium and sodium-ion batteries†

**DOI:** 10.1039/d1na00081k

**Published:** 2021-04-12

**Authors:** Philip A. Maughan, Luc Bouscarrat, Valerie R. Seymour, Shouqi Shao, Sarah J. Haigh, Richard Dawson, Nuria Tapia-Ruiz, Nuno Bimbo

**Affiliations:** Department of Engineering, Lancaster University Lancaster LA1 4YW UK n.bimbo@soton.ac.uk; Department of Chemistry, Lancaster University Lancaster LA1 4YB UK n.tapiaruiz@lancaster.ac.uk; Department of Materials, University of Manchester Manchester M13 9PL UK

## Abstract

In this work, we apply an amine-assisted silica pillaring method to create the first example of a porous Mo_2_TiC_2_ MXene with nanoengineered interlayer distances. The pillared Mo_2_TiC_2_ has a surface area of 202 m^2^ g^−1^, which is among the highest reported for any MXene, and has a variable gallery height between 0.7 and 3 nm. The expanded interlayer distance leads to significantly enhanced cycling performance for Li-ion storage, with superior capacity, rate capably and cycling stability in comparison to the non-pillared analogue. The pillared Mo_2_TiC_2_ achieved a capacity over 1.7 times greater than multilayered MXene at 20 mA g^−1^ (≈320 mA h g^−1^) and 2.5 times higher at 1 A g^−1^ (≈150 mA h g^−1^). The fast-charging properties of pillared Mo_2_TiC_2_ are further demonstrated by outstanding stability even at 1 A g^−1^ (under 8 min charge time), retaining 80% of the initial capacity after 500 cycles. Furthermore, we use a combination of spectroscopic techniques (*i.e.* XPS, NMR and Raman) to show unambiguously that the charge storage mechanism of this MXene occurs by a conversion reaction through the formation of Li_2_O. This reaction increases by 2-fold the capacity beyond intercalation, and therefore, its understanding is crucial for further development of this family of materials. In addition, we also investigate for the first time the sodium storage properties of the pillared and non-pillared Mo_2_TiC_2_.

## Abstract 2

We apply a silica pillaring method to create a porous Mo_2_TiC_2_ MXene with nanoengineered interlayer distances, significantly improving performance in Li and Na-ion batteries. The charge storage mechanism is studied experimentally for the first time.

## Graphical abstract

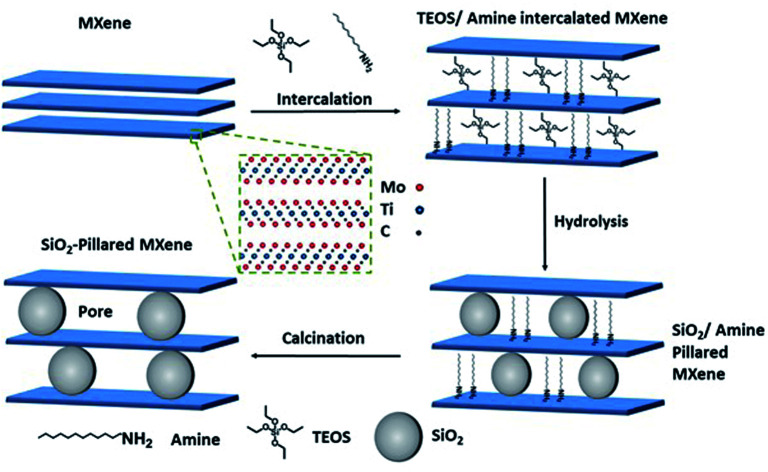

## Introduction

Over recent years, there has been incredible growth in the research and application of Li-ion batteries (LIBs), which are now widely used in portable electronics, electric vehicles and grid storage applications.^1,2^ However, further uptake of these technologies in more demanding applications, such as fast-charging electric vehicles and grid storage, requires significant improvements in high-rate charging and cycling lifetime, without compromising their energy density. Since these characteristics are determined by the electrode materials, there is an urgent need to develop new materials that can satisfy these demands. The negative electrodes that are currently used for Li-ion batteries often suffer from poor rate capability (for example, the state-of-the-art negative electrode material graphite), and those with an impressive performance at high rates cannot achieve high capacities overall (lithium titanate only achieves capacities around 150 mA h g^−1^ and niobium oxides have a capacity of 200 mA h g^−1^.^3–5^

Two-dimensional (2D) materials such as graphene have emerged as promising candidates for next-generation high-rate negative electrode materials due to their combination of high electrical conductivity and large 2D channels, which allow for fast electron and Li^+^ diffusion, respectively, facilitating fast charging times.^6^ However, 2D materials typically suffer from issues such as restacking of nanosheets during cycling, which can block Li diffusion channels, leading to lowered capacities, rate capabilities and cycling stabilities.^7^ It is therefore crucial to develop methods which give rise to controlled open electrode architectures that are stable even when cycled at high rates.

MXenes are an exciting family of 2D materials which have attracted significant research attention since their discovery in 2011, especially in the field of energy storage.^8–11^ They have demonstrated multiple advantageous properties for LIBs such as higher conductivity and large tuneable interlayer spacings. These enable excellent rate capability and cycling stability compared to practical liquid-processed films from other 2D materials such as transition metal dichalcogenides (TMD) and reduced graphene oxides (rGO).^12–14^ In addition, MXenes typically have much higher tap density than TMDs and graphenes, which would result in significantly higher volumetric energy density in batteries.^14^ However, like other 2D materials, electrode architecture plays a crucial role in their electrochemical performance, with multilayered or restacked MXenes showing poor cycling performance.^15,16^ Titanium-based MXenes such as Ti_2_C or Ti_3_C_2_ dominate research in this area, despite more than 30 different MXenes having been synthesised to-date.^17^ This is particularly important because Ti-based MXenes suffer from poor initial coulombic efficiencies (typically 40–60%), which severely limit their application in full cells.^18^ In 2015, Anasori *et al.* first reported Mo_2_TiC_2_, an out-of-plane ordered MXene, with Mo occupying the outer metal layers, while the inner metal layer is exclusively Ti.^19^ This allows the effect of the outer metal element to be studied, since Mo_2_TiC_2_ is otherwise analogous to Ti_3_C_2_.^19^ Despite this, there have only been a handful of reports on this MXene,^19–23^ with only one other reporting LIB performance.^21^ Mo_2_TiC_2_ had several promising features for LIB applications, with delaminated Mo_2_TiC_2_ showing capacities up to 260 mA h g^−1^, an initial coulombic efficiency of 86% and low average voltage.^19^

Unlike Ti-based MXenes, the load curve for Mo_2_TiC_2_ displayed a plateau below 0.6 V, suggesting a different charge storage mechanism compared to MXenes without Mo. Computational studies implied that a conversion reaction occurs between lithiated Mo–O surface groups (formed *via* Li intercalation in reaction (1) and two further moles of Li, as shown by reaction (2)), boosting the capacity.^19^ The theoretical capacity achieved in reaction (1) is 180 mA h g^−1^, which increases to 356 mA h g^−1^ after the proposed conversion reaction.

1 Mo_2_TiC_2_O_2_ + 2Li^+^ + 2e^−^ → Mo_2_TiC_2_O_2_Li_2_

2 Mo_2_TiC_2_O_2_Li_2_ + 2Li^+^ + 2e^−^ → Mo_2_TiC_2_ + 2Li_2_O

The proposed mechanism is similar to the lithiation of Mo oxides, which is accompanied by large volume changes, causing significant capacity fade.^24^ This could explain the relatively high fade seen in previous Mo_2_TiC_2_ studies,^19,21^ demonstrating the need to develop methods that optimise the electrode architecture and increase the cycling stability of this material. There have been no reports to-date on engineered electrode architectures for Mo_2_TiC_2_ in electrochemical applications, despite the clear promise of this material. Furthermore, previous work has not experimentally verified the lithiation mechanism.

Pillaring is a technique used to make porous layered materials from non-porous precursors, by inserting foreign species into the interlayer, which expands the pore space and creates stable architectures which prevent sheets from restacking.^25^ This technique has recently been applied to MXenes, and has been shown to improve performance in a variety of electrochemical energy storage applications such as Li, Na and Zn-ion batteries, aqueous supercapacitors and solid-state supercapacitors.^26–33^ However, these reports have been limited to titanium-based MXenes, with no studies of pillaring having considered the wider MXene family.

**Fig. 1:**
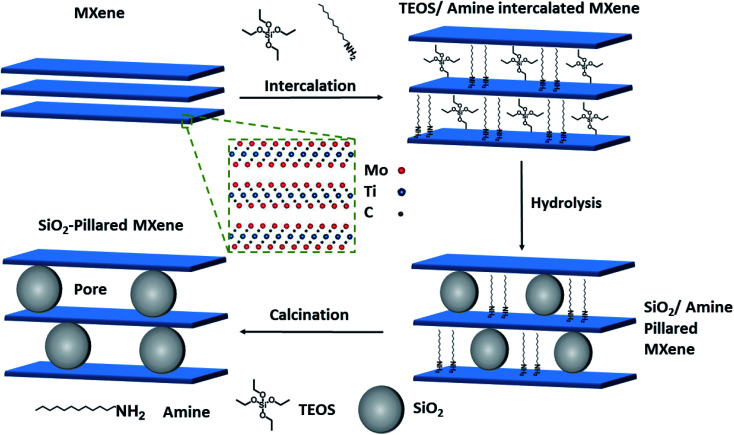
Schematic representation of the amine-assisted silica pillaring method. Inset (dashed green box) illustrates the crystal structure of the out–out-plane ordered Mo_2_TiC_2_ MXene before pillaring. Intercalation with dodecylamine (DDA) and tetraethyl orthosilicate (TEOS) is used to form Mo_2_TiC_2_–Si, followed by hydrolysis and calcination steps to form the pillared material, Mo_2_TiC_2_–Si-400.

In this work, we developed a porous Mo_2_TiC_2_ architecture using an amine-assisted pillaring technique (Fig. 1) and obtained the largest BET surface area reported for any Mo-based MXene to-date. We tested the resulting pillared Mo_2_TiC_2_ for electrochemical Li- and Na-ion storage, and obtained significantly enhanced performance, with superior capacities, rate capabilities and cycling stabilities compared to the non-pillared version, proving the effectivity of this pillaring method. Furthermore, the charge compensation mechanism in Mo-based MXenes was investigated for the first time, using a combination of spectroscopic techniques, including nuclear magnetic resonance spectroscopy (NMR), X-ray photoelectron spectroscopy (XPS) and Raman spectroscopy. We believe that these fundamental studies are crucial for the future development of this class of MXene materials as electrodes in LIBs in order to achieve high-rates and long cycle-life. In addition, due to the growing importance of Na-ion battery research as a promising low-cost alternative to LIBs, we also report for the first time results for the use of Mo_2_TiC_2_ in Na-ion half-cells.

## Results

### MAX phase and MXene synthesis

**Fig. 2:**
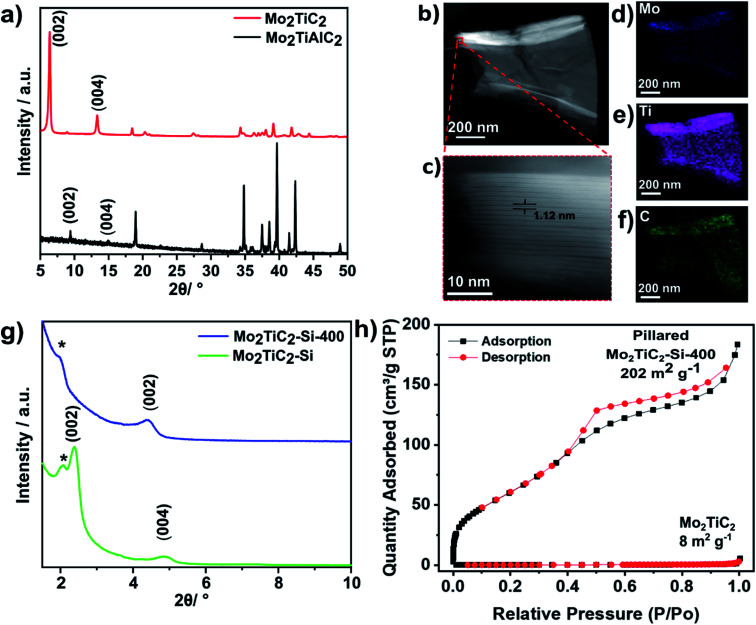
(a) PXRD data of the as-synthesised Mo_2_TiAlC_2_ MAX phase (bottom) and Mo_2_TiC_2_ MXene (top). (b) HAADF-STEM micrograph of Mo_2_TiC_2_. The dashed red square highlights the region shown in the expansion in (b). (c) Expanded HAADF-STEM micrograph of the highlighted area in (b). (d–f) STEM-EDS maps of (b). (g) Low angle PXRD data of the intercalated (Mo_2_TiC_2_–Si) and calcined (Mo_2_TiC_2_–Si-400) pillared MXene materials. (*) denotes a peak from the glass sample holder. (h) N_2_ adsorption–desorption curves at 77 K for the pillared (Mo_2_TiC_2_–Si-400) and non-pillared Mo_2_TiC_2_.

The Mo_2_TiAlC_2_ MAX phase was synthesised following previously reported methods, with details given in the experimental section.^19,34^ The powder X-ray diffraction (PXRD) data matches previous reports, showing the successful synthesis of Mo_2_TiAlC_2_ (Fig. 2a).^19^

To avoid the risk associated with handling HF, the synthesis of Mo_2_TiC_2_ was carried out for the first time using an adapted version of the LiF–HCl method which has been successfully used for titanium-based MXenes.^35^ Details of this can be found in the experimental section. PXRD data are in agreement with previously reported X-ray diffraction data for Mo_2_TiC_2_,^19^ demonstrating that the Mo_2_TiC_2_ MXene was successfully etched using the described method (Fig. 2a). The (002) diffraction peak of Mo_2_TiC_2_ has shifted to a lower angle compared to the MAX phase (to *ca.* 7° 2*θ*), and has increased in intensity, as expected for the formation of a MXene phase.^36^ Both the (002) and (004) diffraction peaks have shifted to higher angles, implying a larger *d*-spacing compared to previously reported HF-etched Mo_2_TiC_2_,^19^ which is consistent with the pre-intercalation of Li during the etching method, as has been observed for Ti_3_C_2_.^35^ A small peak at 9.5° 2*θ*, which corresponds to the (002) diffraction peak of Mo_2_TiAlC_2_, indicates that a minimal impurity corresponding to the original MAX phase remains. High-angle annular dark-field scanning transmission electron microscopy (HAADF-STEM) and energy dispersive X-ray spectroscopy (EDS) were also carried out to further confirm the formation of the Mo_2_TiC_2_ MXene. HAADF-STEM micrographs (Fig. 2b and c) show the layered morphology typical of an MXene in the etched material, with an interlayer spacing *ca.* 1.2 nm, supporting the PXRD results. Scanning electron microscopy (SEM) revealed the flakes have lateral dimensions in the range of 1–10 μm (Fig. S1†), with thicknesses between 1 and 5 μm, which are similar in size to other reported MXenes, including Mo_2_TiC_2_.^19^ SEM-EDS and STEM-EDS analysis show that the MXene consist of homogenously distributed Mo, Ti and C (Fig. 2c–f), with quantification revealing an atomic ratio of approximately 1 : 0.5 : 1 (normalised to Mo), which supports the expected stoichiometry (Fig. S1 and S2†). SEM-EDS confirmed that the flakes contain no Al, demonstrating successful etching of the MAX phase and that Mo_2_TiC_2_ is terminated with –O and/or –OH groups (21 at%) and –F groups (3 at%), akin to its Ti_3_C_2_ counterpart (Fig. S1†). BET analysis (Fig. 2h) using a nitrogen isotherm at 77 K showed the as-made MXene was non-porous, with a low specific surface area of 8 m^2^ g^−1^.

Having confirmed the successful synthesis of the Mo_2_TiC_2_, we then applied our previously reported amine-assisted SiO_2_ pillaring method to this MXene (see Fig. 1 and the Experimental section).^37^ Throughout the paper, we refer to the pillared sample intercalated with dodecylamine (DDA) and tetraethyl orthosilicate (TEOS) as Mo_2_TiC_2_–Si and the pillared sample after calcination at 400 °C as Mo_2_TiC_2_–Si-400.

**Fig. 3:**
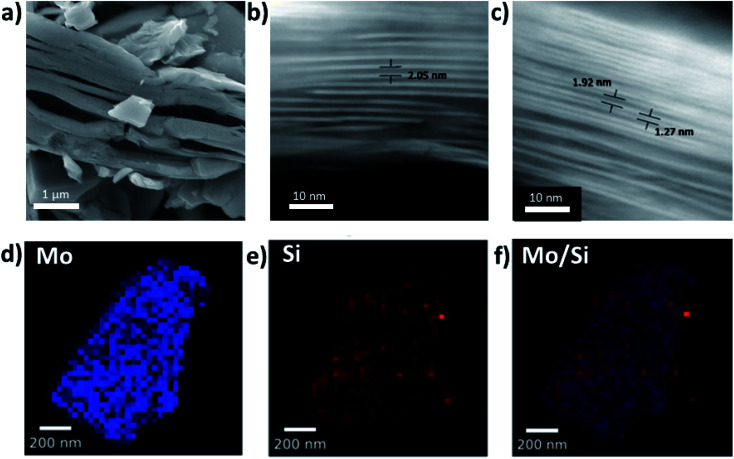
(a) SEM micrograph of Mo_2_TiC_2_–Si-400 MXene after calcination at 400 °C for 5 h under flowing argon. (b and c) HAADF-STEM micrographs of (b) Si intercalated MXene, Mo_2_TiC_2_–Si, and (c) Si-pillared and calcined MXene, Mo_2_TiC_2_–Si-400. (d and f) STEM-EDS maps of Si-pillared MXene, Mo_2_TiC_2_–Si-400 showing the Mo (d) and Si (e) distribution in the pillared MXene, with the overlay shown in (f).

PXRD data show a clear shift in the (002) diffraction peak of Mo_2_TiC_2_–Si from 7 to 2.5° 2*θ*, demonstrating intercalation (Fig. 2g). This gives an increased *d*-spacing of *ca.* 3.6 nm, corresponding to a gallery height of around 2.6 nm, larger than any reported for other layered Mo-based materials such as Mo_2_C and MoS_2_ (Table S1†).^22,38–40^ After calcination (Mo_2_TiC_2_–Si-400), the (002) diffraction peak shifts to a higher angle of 4.5° 2*θ* (Fig. 2g), corresponding to a *d*-spacing of around 2 nm, which gives a gallery height of 1 nm. The shift suggests that the DDA template is successfully removed, which is also supported by the loss of peaks corresponding to DDA in the Raman spectra for this material (Fig. S3†). SEM and STEM studies (Fig. 3a–c) also confirm that the layered morphology of the MXene is retained after calcination, albeit with slightly lower interlayer distances measured compared to the PXRD results (2.04 ± 0.26 nm after intercalation, and 1.50 ± 0.41 nm after calcination from STEM measurements). We have seen such discrepancies between PXRD and STEM data in our previous works.^37^ STEM-EDS results demonstrate significant Si content (*ca.* 2.6 at%) in the final material (Fig. S2†). The Si does not form clusters across the MXene flake, and surface crystals of SiO_2_ are not visible. This supports the formation of nanoscale SiO_2_ pillars between the MXene layers, since Si is shown to be distributed evenly across the flake. Prior to calcination, STEM-EDS mapping (Fig. S4†) showed significant increases in Si concentration at the edges of the flakes, suggesting the initial Si intercalation favours near surface sites in the MXene. The calcination step then allows a deeper intercalation of the Si, increasing homogeneity of the Si distribution, demonstrating the importance of the calcination stage to create a well pillared porous product.

BET analysis revealed that the pillaring procedure resulted in a substantial increase in BET specific surface area, with the pillared Mo_2_TiC_2_ MXene (Mo_2_TiC_2_–Si-400) obtaining a surface area of 202 m^2^ g^−1^ compared to around 8 m^2^ g^−1^ for the non-pillared material (Fig. 2h). This is one of the largest surface areas reported for any MXene, and the largest for a non-Ti based MXene.^15,41–44^ Additionally, this is also larger than previously reported results for pillared MoS_2_ (Table S1†).^40,45^ Pore size distribution analysis on the pillared MXene demonstrated the existence of pores just over 1 nm in size (Fig. S5†), in close agreement with the PXRD data (Fig. 2d), supporting the presence of large interlayer pores in the pillared MXene. For the remainder of the manuscript, pillared Mo_2_TiC_2_ refers to the calcined material Mo_2_TiC_2_–Si-400.

**Fig. 4:**
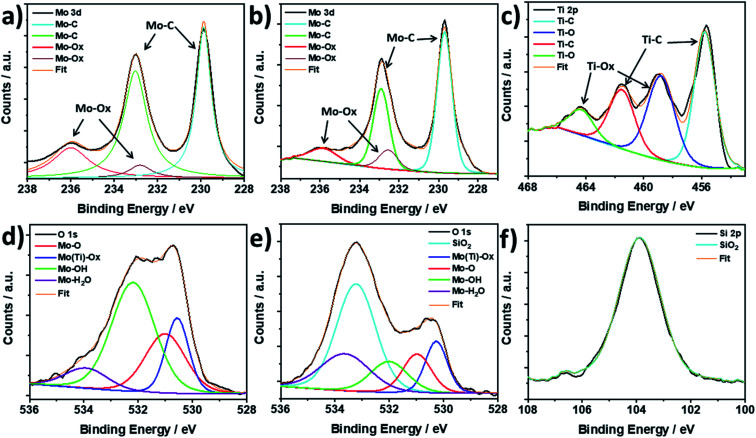
Mo 3d XPS spectra for (a) non-pillared Mo_2_TiC_2_ and (b) Mo_2_TiC_2_–Si-400 after calcination at 400 °C, Mo_2_TiC_2_–Si-400. (c) Ti 2p XPS spectra of Mo_2_TiC_2_–Si-400. (d) O 1s spectra of non-pillared Mo_2_TiC_2_. (e) O 1s spectra of the pillared MXene Mo_2_TiC_2_–Si-400. (f) Si 2p spectra of the pillared MXene Mo_2_TiC_2_–Si-400.

To further investigate the structure of the synthesised Mo_2_TiC_2_ and the effect of the pillaring process on the MXene, X-ray photoelectron spectroscopy (XPS) was used to study the Mo, Ti and O valence states and surface functional groups in the as-made and pillared MXene, and Si was also studied in the pillared material (Mo_2_TiC_2_–Si-400). We note that high resolution XPS is capable is distinguishing between multiple similar species commonly found within MXenes, such surface –O and –OH groups, and intercalated water, and is therefore more powerful than alternative techniques such as infra-red (IR) spectroscopy.^20,46^Fig. 4 shows the Mo 3d XPS spectra for the non-pillared and pillared Mo_2_TiC_2_. There are three main peaks visible in both samples centred at 229.8, 233.0 and 236.0 eV for Mo_2_TiC_2_ and 229.7, 232.9 and 236.1 eV for Mo_2_TiC_2_–Si-400. These peaks are in good agreement with a previous report on Mo_2_TiC_2_ Mo 3d XPS spectra, supporting a successful Mo_2_TiC_2_ synthesis.^20^ It is known that the large peaks at around 229.8 and 233.0 eV correspond to the electrons in the 3d_5/2_ and 3d_3/2_ levels for the expected MXene Mo environment (Mo–C), which indicates that 4^+^ is the dominant oxidation state. The small peak at 236.0 eV corresponds to surface Mo oxides, (Mo^6+^, 3d_3/2_ electrons) showing that, like titanium-based MXenes, Mo_2_TiC_2_ also undergoes a slight surface oxidation either during the etching process or when exposed to ambient conditions.^47^ The Mo^6+^ 3d_5/2_ electrons contribute to the peak centred around 233.0 eV, with an expected binding energy of 232.7 eV. The presence of a small amount of these surface oxides has also been reported previously for Mo_2_TiC_2_, where HF etching was used.^48^ The surface oxide peaks do not appear to grow after pillaring and calcination, suggesting that the Ar atmosphere during calcination was sufficient to avoid further oxidation of the MXene. Ti 3d XPS spectra (Fig. 4c and S5†) follows the same pattern, with 3^+^ being the dominant oxidation state and some Ti^4+^ oxides also present.

XPS results for the O 1s scans show clear differences between the non-pillared and pillared Mo_2_TiC_2_ (Fig. 4d and e, respectively). The spectrum for the non-pillared Mo_2_TiC_2_ matches previous reports for this MXene and shows a very broad asymmetric peak which is comprised of a variety of components as a result of multiple oxygen-containing species being present in the sample.^20^ Peak deconvolution reveals a component centred around 530.5 eV, which corresponds to the formation of Mo and Ti oxides, supporting the Mo 3d and Ti 2p spectra.^20^ The peak at 531.0 eV corresponds to Mo–O groups, while the peak around 532.0 eV reveals the presence of Mo–OH termination groups.^20^ At 534.0 eV there is a small component which corresponds to surface-bound H_2_O molecules.^20^ After pillaring and calcination, there is a significant new broad peak centred around 533.2 eV, which is a result of oxygen in the silica pillars.^49^ There is a substantial decrease in the component relating to –OH surface groups. Before pillaring, the OH : O ratio is approximately 2 : 1, as can be seen in 4d, but decreases significantly to 1 : 1 after pillaring (4e), demonstrating the direct involvement of the –OH groups in the pillaring process, as we have reported in our previous work for Ti_3_C_2_.^37^ Finally, the Si 2p XPS spectra for Mo_2_TiC_2_–Si-400 (Fig. 4f) shows a broad peak at 103.9 eV, which is consistent with SiO_2_ being the pillar.^49^

Overall, these results imply that the amine-assisted pillaring method is unaffected by the change in metal in the surface layer of the MXene, and is directly applicable to Mo-based MXenes. This suggests that the pillaring method can be applied to other types of MXenes, so long as –OH surface groups are present.

### Electrochemical testing in a Li-ion battery

**Fig. 5:**
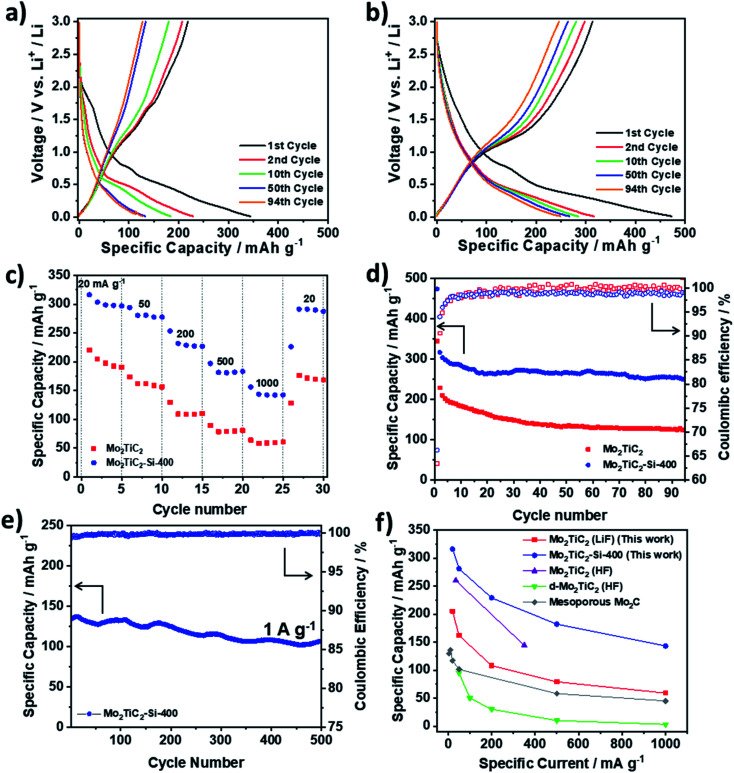
Galvanostatic charge–discharge testing of Mo_2_TiC_2_ samples in Li-ion half-cells in a voltage range of 0.01–3 V *vs.* Li^+^/Li. (a) Load curves for selected cycles of Mo_2_TiC_2_. (b) Load curves for selected cycles of Mo_2_TiC_2_–Si-400. (c) Rate capability testing at 20, 50, 200, 500, 1000 mA g^−1^ for five cycles at each rate for the pillared (blue) and non-pillared (red) samples. (d) Cycling stability data and coulombic efficiencies over 94 cycles for the pillared (blue) and non-pillared (red) samples. (e) Long-term high-rate cycling stability of Mo_2_TiC_2_–Si-400 at 1 A g^−1^ over 500 cycles. (f) Rate capability comparison of the performance of the Mo_2_TiC_2_ MXenes reported in this work with existing reports of Mo-based MXenes electrodes supported on Cu current collectors for lithium-ion batteries: Mo_2_TiC_2_ (HF),^19^ d-Mo_2_TiC_2_ (HF)^21^ and mesoporous Mo_2_C.^51^

To evaluate the electrochemical performance of the pillared structure, the Mo_2_TiC_2_ materials were tested in half-cells against Li metal, which acts as a counter and reference electrode. Fig. 5 shows galvanostatic cycling data in a voltage window of 0.01–3 V *vs.* Li^+^/Li at a rate of 20 mA g^−1^. The first cycle capacities for the pillared MXene are 473 and 314 mA h g^−1^ on the discharge and charge respectively (Fig. 5b), which are larger than for the non-pillared MXene (344 and 219 mA h g^−1^ respectively, Fig. 5a), and previously reported Mo_2_TiC_2_ (Table S2†).^19,21^ The low coulombic efficiency of *ca.* 66% in the first cycle (Fig. 5) is commonly observed in MXenes and is attributed to SEI formation and irreversible reactions between surface groups and Li^+^ ions.^13,50^ This is lower than a previous report for Mo_2_TiC_2_ (86%),^19^ which is likely explained by higher levels of SEI formation in the pillared MXene as a result of the significantly larger surface area, which cancels out any improvements obtained by reducing ion trapping by the removal of narrow interlayer sites. Differences in surface chemistry between the materials may also explain the variation. The coulombic efficiency in the second cycle is 94% and reaches *ca.* 99% after 18 cycles in both MXenes. Around 80% capacity is retained between the 2^nd^ (316 mA h g^−1^) and 94^th^ (250 mA h g^−1^) cycles in the pillared MXene (Fig. 5d) compared to just 54% capacity retention under the same conditions for the as-made Mo_2_TiC_2_. Chen *et al.* reported a capacity of only 52 mA h g^−1^ by the 100^th^ cycle in their tests on Mo_2_TiC_2_,^4^ which further demonstrates the remarkable improvement in cycling stability afforded by the pillaring technique reported here.

Rate capability tests were carried out at increasing rates of 20, 50, 200, 500, and 1000 mA g^−1^ with five discharge–charge cycles at each rate (Fig. 5c). The pillared material shows superior performance at all rates, delivering discharge capacities of 312, 281, 229, 182 and 143 mA h g^−1^, respectively. When the current was returned to 20 mA g^−1^, the capacity was recovered to 292 mA h g^−1^. In comparison, the non-pillared Mo_2_TiC_2_ material delivered capacities of 205, 162, 108, 79 and 59 mA h g^−1^ at the respective rates, with 172 mA h g^−1^ recovered at 20 mA g^−1^. Notably, the enhancement in capacity between the pillared and non-pillared MXene increases with rate, with the pillared MXene delivering capacities around 1.7 times greater than for the non-pillared MXene at 20 mA g^−1^, and around 2.5 times greater than for the non-pillared MXene at 1 A g^−1^. This demonstrates that the increased interlayer spacing afforded by the pillaring enables fast Li-ion transport, resulting in superior capacities at higher rates.

Since the pillared MXene showed impressive capacity at high rates, its high-rate cycling stability was then tested by continuous galvanostatic cycling at 1000 mA g^−1^ (corresponding to a charging/discharging time of 8 min) after the rate capability test. After 500 cycles at 1000 mA g^−1^, it retained a capacity of 108 mA h g^−1^, a capacity retention of 80% compared to the 1st cycle (135 mA h g^−1^), (Fig. 5e). The average coulombic efficiency over these cycles was close to 100%, indicating highly reversible charge storage at this rate. This shows that Mo_2_TiC_2_–Si-400 is a very stable electrode, making it highly suitable for high-power and long-life batteries. In addition, a comprehensive comparison of our work with other Mo-based MXenes for LIB applications demonstrates the superior performance of our pillared Mo_2_TiC_2_ material (Fig. 5f and Table S2†).

The load curves for both materials, (*i.e.* non-pillared and pillared, Fig. 5a and b, respectively) display very similar features and are markedly different from titanium-based MXenes, which typically display very linear profiles.^52^ In both materials the load curves show two clear regions on discharge after the first cycle, the first of which is between 3 and 0.6 V, which slopes with a linear profile and a second region between 0.6 and 0.01 V where there is a sloping plateau feature appearing, demonstrating that a different charge storage mechanism operates in this region. Closer inspection of the load curves reveals that the majority of the capacity is stored in the region below 0.6 V (215 mA h g^−1^ for the pillared material on the 2nd cycle), but that the capacity fade also occurs mostly in that region (160 mA h g^−1^, 73%, is retained after 94 cycles for the pillared material, Mo_2_TiC_2_–Si-400). This capacity loss is even more dramatic in the non-pillared MXene, where the sloping plateau feature is substantially reduced during cycling to just 90 mA h g^−1^ after 94 cycles (53% capacity retention). Furthermore, differential d*Q* d*V*^−1^ plots of both non-pillared and pillared samples (Fig. S7 and S8†) show a peak at 0.6 V, which rapidly decreases with cycling, confirming that this low voltage process contributes significantly to the capacity during the initial cycles but it is a significant cause of capacity fade over prolonged cycling.

Anasori *et al.* used density functional theory (DFT) to predict that the 0.6 V feature present in the load curves of Mo_2_TiC_2_ could result from a conversion reaction between Mo–O groups on lithiated Mo_2_TiC_2_O_*x*_ (formed by Li intercalation between 3 and 0.6 V) and extra Li moles to form Li_2_O.^19^ Li_2_O is known to be a poor electrical conductor, and it is possible that its formation, which in transition metal oxide electrodes is often poorly reversible and accompanied by a large volume change, is the main cause of the capacity fade seen in these electrodes, with several bulk oxides reporting capacity retention values between 40–50% over 100 cycles.^53^ In these reports on Mo oxides, Li_2_O typically forms amorphous nanosized particles which cannot be observed using techniques which rely on long-range order such as XRD.^54–56^ Therefore, we used a series of spectroscopic studies (NMR, XPS and Raman) to experimentally validate the charge storage mechanism for the first time. These techniques are able to probe the local structure and surface environments of materials, and are typically utilised to investigate the formation of Li_2_O as a discharge product in Mo oxides.^54,55^ To ensure samples were not degraded by exposure to air, all electrodes were extracted at the desired state-of-charge in an argon or nitrogen filled glovebox, washed with dimethylcarbonate (DMC) to remove any SEI components and electrolyte salts and sealed in air tight containers for transport/measurement.

**Fig. 6:**
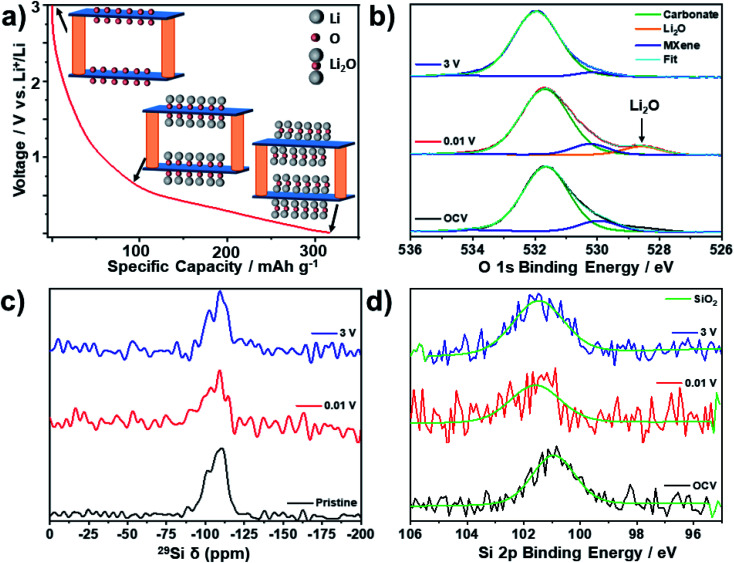
*Ex situ* MAS SS-NMR spectra (16.4 T, 30 kHz MAS) and XPS spectra of Mo_2_TiC_2_–Si-400 at selected states-of-charge. (a) Schematic illustrating the lithiation mechanism for oxygen-terminated Mo_2_TiC_2_, with the MXene sheets shown in blue and the pillars in orange. (b) *Ex situ* O 1s XPS spectra. (c) *Ex situ*^29^Si MAS SS-NMR spectra. (d) *Ex situ* Si 2p XPS spectra.

To study the reactivity of the SiO_2_ pillars, we combined ^29^Si NMR and Si 2p XPS data to investigate any potential lithiation of the pillars, both in the bulk and near the surface of the pillared MXene. ^29^Si solid-state NMR (Fig. 6c) revealed that there is only one Si environment present within the pristine pillared MXene structure, which matches well with the expected chemical shift for SiO_2_ (*i.e.* −108 ppm).^57^ There is no change in the ^29^Si NMR environment after discharge or subsequent charge, confirming that the SiO_2_ pillars are stable during cycling, and that no alloying reaction occurs. This is supported by *ex situ* Si 2p XPS (Fig. 6d), which also shows no significant changes in the spectra at different states-of-charge, suggesting no difference in redox activity between bulk and near-surface pillars. Additionally, no signal corresponding to Li_*x*_Si_*y*_ alloys (expected between 20 and 10 ppm) could be distinguished in the ^7^Li NMR spectra (Fig. S9†).^57,58^ Significantly, this means that the improved electrochemical performance seen in the pillared Mo_2_TiC_2_ is a result of the enlarged interlayer spacing, and not due to the lithiation of SiO_2_.

*Ex situ* O 1s XPS analysis was used to investigate the proposed mechanism of reversible Li_2_O formation in Mo_2_TiC_2_ (Fig. 6b). At OCV there is just one broad peak visible, which is centred on 532.0 eV. Peak deconvolution reveals that there are two main components to this peak at 531.7 eV (assigned to the carbonate oxygen from the electrolyte)^59^ and 530.6 eV (which matches the Mo–O_*x*_ environment present in the powdered Mo_2_TiC_2_ material in Fig. 4). There is little change in these two peaks upon cycling, with the shifts varying less than 0.2 eV at all states-of-charge. However, the spectrum for the electrode discharged to 0.01 V shows a new peak at 528.6 eV, which matches well with Li_2_O.^49^ Upon charging to 3 V, this peak disappears, confirming that Li_2_O is reversibly formed and removed upon lithiation (discharging) and delithiation (charging) in the pillared material.

*Ex situ* solid-state NMR (SS-NMR) was then used to study the evolution of the local structure of the H, Li and F environments within the bulk of the pillared MXene upon cycling. ^1^H NMR showed a broad peak present in the pristine sample, which is assigned to ^1^H environments in the polyvinylidene fluoride (PVDF) binder (Fig. S10†).^60^ After discharge to 0.01 V, new peaks appear at 3 and 4 ppm, which correspond to carbonate environments originating from retained electrolyte solvent molecules in the material.^60^ In addition, a new peak at −1.5 ppm is now present, which can clearly be assigned as LiOH based on previous reports.^60^ This peak disappears after charging to 3 V, suggesting that, like Li_2_O, the formation of LiOH is reversible. This implies that LiOH is a discharge product, rather than a component of the SEI, which would be expected to still be present upon charging. In addition, all extracted electrodes were washed with DMC to remove SEI and electrolyte residues.

Overall, these results support the mechanism proposed by Anasori *et al.* (reactions (1) and (2)),^19^ but imply that an additional reaction involving the formation of LiOH, most likely *via* a conversion reaction with terminal –OH groups, may also occur (reaction (3)).

3 Mo_2_TiC_2_(OH)_2_ + 2Li^+^ + 2e^−^ → Mo_2_TiC_2_ + 2LiOH

This reaction could explain the second low voltage discharge peak observed in the d*Q* d*V*^−1^ plots for both the non-pillared and pillared Mo_2_TiC_2_ (Fig. S7 and S8†). An analogous conversion reaction involving the formation of LiOH has been previously observed on RuO_2_.^60^ Comparing the stoichiometries of reactions (1) and (2) with reaction (3) suggests that –OH groups may only provide half as much capacity as –O groups.

*Ex situ* Raman spectroscopy supports the proposed conversion reactions (Fig. S11†), showing a reduction in the intensity of the Mo–O Raman modes at 170 and 270 cm^−1^ after discharging to 0.01 V, suggesting the cleavage of these bonds.^21^ It should be noted that some partial re-oxidation is expected, which may explain the continued presence of the 260 cm^−1^ Raman mode. However, LiOH is also known to have a minor Raman mode at 270 cm^−1^,^61,62^ and thus, it could be contributing to the 260 cm^−1^ peak. In addition, LiOH has a major Raman mode at 320 cm^−1^, which could explain the increase in intensity in this region after discharge.^61,62^ However, these modes cannot be conclusively assigned due to the overlap with potential MXene peaks. After discharge, a new mode at *ca.* 550 cm^−1^ matches previous reports for Li_2_O, supporting the formation of Li_2_O as a discharge product.^61,62^ Therefore, these results could support the proposed conversion reaction mechanism whereby the –O and –OH surface functional groups of Mo_2_TiC_2_ MXene react with Li to form Li_2_O and LiOH respectively. Crucially, the Raman spectra confirm that the MXene bonding framework between Mo, C and Ti is unchanged upon cycling despite the conversion reactions unlike with transition metal oxides.^53^ This should ensure superior reversibility during the lithiation process of Mo-based MXenes compared to the metal oxides, as suggested by our cycling data.

^7^Li NMR (Fig. S12†) shows a broad asymmetric peak centred at −0.6 ppm. Deconvolution of this peak using a Lorentzian profile shape for the pristine sample reveals two main environments, the largest of which is assigned as pre-intercalated Li as a result of the LiF–HCl etching method used.^16^ A minor peak to the right of this, centred around −1.1 ppm, can be assigned as LiF from the etching stage, which is confirmed as present in the structure by ^19^F NMR and ^7^Li–^19^F HETCOR NMR (Fig. S13†).^63^ Upon discharge to 0.01 V, the broad peak shifts to a higher chemical shift (centred at −0.1 ppm), with asymmetry now present on the left side of the peak. This reveals the existence of new Li environments, which are likely to be Li_2_O and LiOH based on the previously discussed XPS (Fig. 6b) and ^1^H NMR (Fig. S10†) data and the relative shifts compared to LiF (3–4 ppm higher than the LiF component).^64^ However, the broad profile of the signal, resulting from the slightly disordered nature of MXenes and the lack of separation between the ^7^Li chemical shift of the different environments,^63,64^ means that the environments cannot be unambiguously distinguished in the broad spectra obtained using ^7^Li NMR. Nevertheless, the ^7^Li NMR spectra appear to support the XPS and ^1^H NMR results, confirming the contribution of conversion reactions to the charge storage mechanism. After charging, these changes are reversed, which demonstrates the reversible (de)lithiation of the Mo_2_TiC_2_ MXene.

Cyclic voltammetry (CV) was used to investigate the reactions and kinetics of the system in more detail. Fig. S14† shows the cyclic voltammograms for five cycles collected at a scan rate of 0.2 mV s^−1^ between 0.01–3 V *vs.* Li^+^/Li. Both the non-pillared and pillared MXenes display similar CV features, with small cathodic redox peaks observed at 1.7 and 1.3 V and a large peak below 0.6 V. Above 0.6 V, the voltammograms are fairly rectangular for both materials, indicative of a capacitive-like contribution to the charge storage in this voltage range. On the first cycle, some additional cathodic peaks are observed on discharge at 1.75 and 0.7 V, while the feature below 0.6 V is also more pronounced than on subsequent cycles. These match the extra features observed in the load curves on the first discharge, and are ascribed to irreversible reactions such as SEI formation and Li trapping.^13,50^ This helps explain the large initial irreversible capacity loss present in the load curves on the first cycle (Fig. 5).^13,50^ After the first cycle, the shape of the CV plots do not notably change, apart from a clear decrease in the current below 0.6 V for the non-pillared material, demonstrating the greater fade observed in this material compared to the pillared MXene, which agrees well with the galvanostatic charge–discharge tests (Fig. 5). The large peak below 0.6 V on discharge and the peak at 1.3 V on charge also match well with the plateaus observed on the load curves (Fig. 5) and d*Q* d*V*^−1^ plots (Fig. S8†), and with previous reports on Mo_2_TiC_2_.^19^

To investigate the kinetics of the system in more detail, the cells were then cycled at increasing scan rates of 0.5, 2 and 5 mV s^−1^ (Fig. S15†). As the scan rate increases, both materials show no major changes, with only broadening and small shifts of their redox peaks to lower voltages, suggesting that the majority of the redox reactions are kinetically favoured. The voltammogram run at 5 mV s^−1^ is much more rectangular in shape for Mo_2_TiC_2_–Si-400 compared to the non-pillared Mo_2_TiC_2_, with increased current above 1 V. This is indicative of a greater contribution from capacitive charge storage as a result of the higher interlayer spacing and surface area in the pillared MXene, and explains the enhanced high-rate performance of this material.

This is further supported by analysing the proportion of diffusion-limited (battery-like) and surface-limited (capacitive-like) processes to the overall current. It is well known that the relationship between current and scan rate is proportional to the power half when current is diffusion-limited, whereas the relationship is linear (power of 1) when current is surface-limited.^65^ This allows the formation of a simple power law to determine the proportion of current arising from diffusion or surface limited processes in a mixed mechanism system, as shown by eqn (4), where *i* is current, *ν* is scan rate and *a* and *b* are fitting parameters.^65^ Plotting the log of the current against the log of the scan rate gives a straight line with a gradient of *b*, allowing the proportion of diffusion (*b* = 0.5) or surface (*b* = 1) limited current to be quantified.

4 *i* = *av*^*b*^

When this analysis is carried out at different voltages, the relative contribution of these processes can be studied across the voltage window on the charge and discharge sweeps (Fig. S15c and d†). For both materials, the *b*-values are closer to 1 (capacitive current) at higher voltages and to 0.5 (diffusion-limited battery-like processes) at low voltages (below 0.6 V). This is expected from the CV shapes, which are more rectangular at higher voltages, with prominent redox peaks present at voltages below 0.6 V. At higher voltages, the pillared Mo_2_TiC_2_ has a higher *b*-value (*i.e.* 0.86) than the non-pillared MXene (0.6–0.8) indicting an increased capacitive contribution to the charge storage resulting from the larger surface area, as previously discussed. In contrast, at low voltages, for example, 0.01 V, the pillared Mo_2_TiC_2_ has a lower *b*-value (0.56) than the non-pillared Mo_2_TiC_2_ (0.68), which indicates a greater contribution from battery-like processes at these voltages. This suggests that pillaring leads to increased charge storage contribution from the Li_2_O conversion reaction, which explains the substantial increases in capacity compared to the non-pillared MXene.

### Electrochemical performance in a sodium-ion battery

**Fig. 7:**
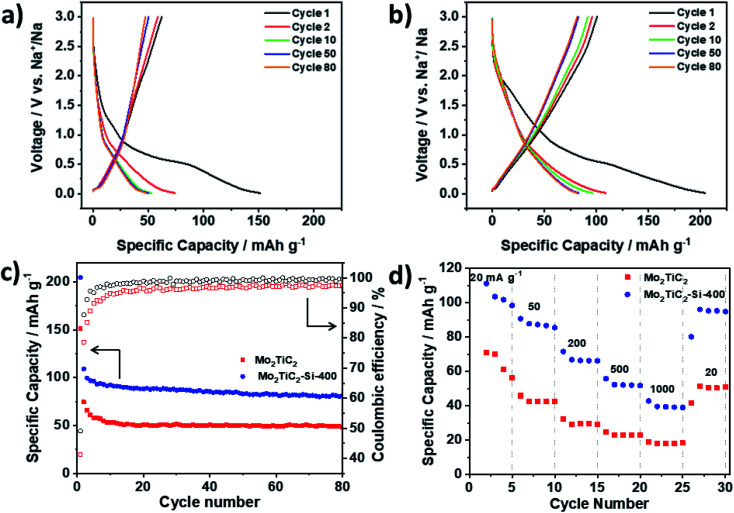
Galvanostatic discharge–charge load curves of Mo_2_TiC_2_ samples in Na-ion half-cells in the potential range of 0.01–3 V *vs.* Na^+^/Na. (a) Load curves at 20 mA g^−1^ for selected cycles of Mo_2_TiC_2_. (b) Load curves at 20 mA g^−1^ for selected cycles of Mo_2_TiC_2_–Si-400. (c) Cycling stability data and coulombic efficiencies over 80 cycles for the pillared (Mo_2_TiC_2_–Si-400, blue) and non-pillared (Mo_2_TiC_2_–Si-400, red) samples at 20 mA g^−1^. (d) Rate capability tests for the pillared (Mo_2_TiC_2_–Si-400, blue) and non-pillared (Mo_2_TiC_2_–Si-400, red) samples at increasing rates of 20, 50, 200, 500 and 1000 mA g^−1^ for five cycles each.

Following the promising performance of the Mo_2_TiC_2_ MXene in a Li-ion system, the non-pillared and pillared materials were further tested as electrodes in Na half-cells. Mo_2_TiC_2_ has so far not been reported as an electrode for Na-ion batteries. It can be seen that pillaring substantially improves the electrochemical performance, with a 2nd cycle discharge capacity of 109 mA h g^−1^ compared to 74 mA h g^−1^ for the non-pillared material (Fig. 7). By the 80th cycle, the non-pillared MXene had retained a capacity of just 48 mA h g^−1^ (65% capacity retention compared to the 2nd cycle) compared to 82 mA h g^−1^ for the pillared MXene (75% capacity retention). These capacities are much lower than what was observed for the Li-ion system and correspond to a discharge product of approximately Mo_2_TiC_2_O_2_Na (theoretical capacity = 90 mA h g^−1^), suggesting insertion of one mole of Na per formula unit, even in the pillared MXene. Unlike in the Li-ion system, differential d*Q* d*V*^−1^ plots show no peaks associated with a conversion reaction for either the pillared or non-pillared Mo_2_TiC_2_ (Fig. S16†). This suggests that Na_2_O does not form, which is in agreement with the DFT studies of Anasori *et al.*^19^ At higher rates (Fig. 7d), the pillared MXene has superior capacities compared to the non-pillared material at each rate studied, retaining 40 mA h g^−1^ at 1 A g^−1^, compared to 16 mA h g^−1^ for the non-pillared MXene.

These findings explain the differences in the behaviour of Mo_2_TiC_2_ in the Li and Na-ion battery systems. In the Li-ion system, at low voltages the conversion reaction to Li_2_O provides a large amount of extra capacity. In contrast, no such conversion reaction occurs in the Na-ion system, which leads to improved cycling stability (in the non-pillared materials), but with capacities around a third of the Li-ion system, even when pillared. CV analysis at different rates reveals that a much greater proportion of the current is surface controlled (capacitive) compared to the Li-ion system (Fig. S17†), for example Mo_2_TiC_2_–Si-400 has a *b*-value of 0.73 at 0.01 V in the Na-ion system, but 0.56 in the Li-ion system at the same voltage. In the Na system, at voltages above 1 V, the *b*-value for the pillared material is around 0.9, showing that capacitive contributions dominate at higher voltages. This leads to reasonable rate capability (Fig. 7d), with over 40 mA h g^−1^ being retained even at the high rate of 1 A g^−1^, which is higher than any other report for Mo-based MXenes in Na-ion systems (Table S3†).

## Conclusions

Overall, we demonstrate the application of an amine-assisted pillaring method to create porous Mo_2_TiC_2_. This leads to a large increase in interlayer spacing, achieving *d*-spacings up to 4.2 nm. This corresponds to a gallery height (pore size between layers) of around 3 nm before calcination, which is by far the largest for a Mo-based MXene, and larger than any reports found for other Mo-based layered materials such as MoS_2_. This suggests that the amine-assisted silica pillaring method could be applied to a wide range of MXenes, and perhaps other layered materials, if sufficient –OH groups are present on the surface to bind to the amine. Calcination removes the DDA template and reduces the gallery height to a still expanded 1 nm in the final pillared material.

When tested as the negative electrode in a lithium-ion battery, the pillared material showed significantly improved electrochemical performance with respect to the non-pillared material, reaching capacities of up to 316 mA h g^−1^ (89% of the reported theoretical capacity, *i.e.* 356 mA h g^−1^) based on two moles of Li^+^ ions intercalating per formula unit followed by two further moles of Li^+^ ions undergoing a conversion reaction. This is the highest capacity reported so far for Mo_2_TiC_2_. The partially reversible formation of Li_2_O during cycling was confirmed by *ex situ* NMR and XPS studies, while *ex situ* NMR suggests that SiO_2_ does not undergo redox activity during cycling. Therefore, the enhanced electrochemical performance may be ascribed to the enlarged interlayer spacing. Pillaring increases the capacity and cycling stability of the MXene by providing free space for the formation of Li_2_O and intercalation of extra Li ions without allowing layers to restack. In addition, the rate capability is also significantly enhanced, since the enlarged interlayer spacing aids the Li^+^ diffusion to the MXene active site. The pillared material shows high coulombic efficiency (*ca.* 100%) and good stability even at a high rate of 1 A g^−1^ (under 8 min charge time) returning 80% of the initial capacity (135 mA h g^−1^) after 500 cycles. These findings are crucial for the development of other MXenes which are proposed to undergo conversion reactions during lithiation such as V, Ta and Cr chemistries.^66^ A potential parallel conversion reaction forming LiOH from –OH surface groups was implied by *ex situ*^1^H NMR and Raman results, but requires further detailed mechanistic studies to confirm the origin of any LiOH formed.

In a Na-ion system, the capacities of the non-pillared and pillared Mo_2_TiC_2_ MXene were up to a third lower than for the Li-ion system, which could be explained by the charge storage relying on Na^+^ intercalation and capacitance, with no conversion reaction occurring. Nevertheless, the pillared material had superior performance compared to the non-pillared MXene, delivering reversible capacities up to 109 mA h g^−1^ at 20 mA g^−1^ over twice that of the non-pillared MXene.

## Experimental methods

### Materials synthesis and pillaring

For the synthesis of Mo_2_TiAlC_2_, Mo (−325 mesh, 98% purity, Sigma Aldrich), Ti (−325 mesh, 99% purity, Alfa Aesar), Al (−100 + 325 mesh, 99.5% purity, Alfa Aesar), and C (graphite, <20 μm, 99% purity, Sigma Aldrich) powders were mixed with a pestle and mortar in a 2 : 1 : 1.1 : 2 molar ratio. The mixture was then heated in a tube furnace under flowing argon at 1600 °C for 4 h, with a heating rate of 5 °C min^−1^. The resulting block was then crushed with a pestle and mortar and ground to give a fine grey powder.

Typically, 3 g of Mo_2_TiAlC_2_ were slowly added to 30 ml of 9 M HCl with 3 g of pre-dissolved LiF. The mixture was heated to 60 °C and stirred for 5 days. The powder was recovered by centrifuging cycles, with DI water added after each cycle until the pH ≈ 6. The sample was then analysed by PXRD, which showed that significant amounts of unetched MAX phase remained in the sample. Therefore, the partially etched sample was re-dispersed in a fresh etching solution using the same volumes and concentrations used previously. After four days, the solid was collected *via* centrifuging, using the same protocol as described above. A washing step, where the powder was dispersed in 1 M HCl for 3 h at ambient temperature, was used to remove any salt impurities resulting from the etching step. A NaOH washing step was also attempted, but this caused the dissolution of the majority of the powder in approximately 30 min, showing that the Mo_2_TiC_2_ material is not stable in alkali conditions, in contrast to Ti_3_C_2_. Leaving the MAX phase to etch for nine consecutive days with no replenishing of the etching solution does not provide a well etched material, even when 12 M HCl is used with 6 g of LiF (the total amount used when the solution is replenished).

400 mg of the etched MXene was then added to a mixture of dodecylamine (DDA) dissolved in TEOS under argon (1 : 10 : 20 MXene : DDA : TEOS molar ratio). This was stirred in a glass vial sealed under argon at ambient temperature for 4 h. The product was then recovered by vacuum filtration and dried on filter paper under vacuum before being re-dispersed in DI water at ambient temperature for 18 h. The intercalated hydrolysis product was then recovered by vacuum filtration and dried overnight at 60 °C, before calcination at 400 °C for 2 h with a heating rate of 5 °C min^−1^ under flowing argon (due to the reactivity of Mo_2_TiC_2_ with air, Fig. S18†) to give the final pillared Mo_2_TiC_2_.

### Material characterisation

Powder X-ray diffraction (PXRD) was carried out in a Smartlab diffractometer with a 9 kW rotating anode (Rigaku, Tokyo, Japan) using Cu Kα radiation (wavelength of 1.54051 Å) operating in reflection mode with Bragg–Brentano geometry. Prior to PXRD characterisation, all samples were dried in a heated oven at 80 °C for 18 h. The powders were then ground and placed on a glass sample holder and pressed flat with a glass slide.

Scanning electron microscopy (SEM) was performed in a JEOL JSM-7800F (JEOL, Tokyo, Japan), and energy-dispersive X-ray spectroscopy (EDS) was carried out using an X-Max50 (Oxford Instruments, Abingdon, UK) with an accelerating voltage of 10 kV and a working distance of 10 mm. The dried powder samples were dry cast onto a carbon tape support, which was placed on to a copper stub for analysis.

High-angle annular dark-field scanning transmission electron microscopy (HAADF-STEM) was performed in an FEI Titan G2 80–200 ‘ChemiSTEM’ operated at 200 kV at room temperature. STEM-EDS data was acquired with the Titan's Super-X detector system. Hyperspy was used to process all STEM-EDS data, and quantification was performed using the Cliff–Lorimer method with standardless *k*-factors. Each specimen was crushed using a mortar and pestle, dispersed in methanol, drop-cast onto holey carbon-coated copper grids, and dried in a vacuum system (∼10^−5^ mbar) at 100 °C for 2 h. STEM cross-sectional imaging was used to measure 100 individual interlayer spacings for each sample for the interlayer distance calculations.

Gas sorption isotherms were measured on a Micromeritics 3 Flex 3500 gas sorption analyser using high purity nitrogen gas at 77 K. BET surface areas were calculated over a relative pressure range of 0.05–0.15*P*/*P*_0_. Pore size distribution analysis was calculated using the NLDFT (non-linear density functional theory) method with a slit pore model using 3Flex Micromeritics software.

Raman spectroscopy was carried out on a Horiba Lab Raman Spectrometer (Horiba, Minami-ku Kyoto, Japan) with an EM-cooled Synapse camera. Spectra were collected using a 100×, 0.90NA microscope objective. For each measurement, three scans were collected, with a total measurement time of 30 min. The dried powder was sandwiched between two glass microscope slides which were pressed together to give flat MXene particles. One of these slides was then discarded, with the other slide placed flat under the diode green laser (532 nm, 200 μW, 1% intensity) for measurements. For *ex situ* measurements, the extracted discharged electrodes were washed with dimethyl carbonate (DMC), dried in the anti-chamber of an argon-filled glovebox, and transported to the spectrometer in a sealed container. The spectra were collected under air.

### Electrochemical characterisation

Pillared and non-pillared Mo_2_TiC_2_ were tested in coin cells (CR2032 type) in a half-cell configuration using lithium or sodium metal (Tob Energy, China) disks as the counter and reference electrodes and 1 M LiPF_6_ or NaPF_6_ in EC/DEC (1 : 1 weight ratio, 99% purity, Gotion) as the electrolyte. The MXene (active material) was mixed with carbon black (super P) as a conductive additive and PVDF as the binder in a 75 : 15 : 10 weight ratio respectively. The mixture was added to a few ml of *N*-methyl-2-pyrrolidone (NMP, 99.5% purity, Alfa Aesar) to make a slurry, which was then cast onto a Cu foil used as current collector, from which electrodes with a diameter of 16 mm were punched. The active mass loading of each electrode was *ca.* 3.2 mg cm^−2^. Coin cells were constructed in an argon-filled glovebox (O_2_ and H_2_O levels <0.1 ppm) using Whatman micro glass fibre paper as the separator. Galvanostatic tests were carried out on a Neware battery cycler (Neware Technology Ltd, China) at a current density of 20 mA g^−1^ in the potential range of 0.01–3 V *vs.* Li^+^/Li for 94 cycles for the tests in the Li-ion half-cells and in the potential range of 0.01–3 V *vs.* Na^+^/Na for 80 cycles for the tests in the Na-ion half-cells. For rate capability tests, the cells were cycled at a current density of 20 mA g^−1^ for 1 cycle to stabilise the cell before 5 cycles were run at each current density of 20, 50, 200, 500 and 1000 mA g^−1^ before returning to 20 mA g^−1^. For the long-term high-rate cycling test on the pillared MXene, 500 cycles were run at a rate of 1 A g^−1^ after the rate capability test. Cyclic voltammetry (CV) measurements were conducted using an Ivium potentiostat (Ivium Technologies BV, The Netherlands) with increasing scan rates of 0.2, 0.5, 2 and 5 mV s^−1^ for 2 cycles at each rate in the potential range of 0.01–3 V *vs.* Li^+^/Li and Na^+^/Na for the tests in Li-ion and Na-ion half-cells, respectively. In each case, the final cycle at each scan rate was used to calculate the *b*-values.

#### *Ex situ* X-ray photoelectron spectroscopy (XPS)

Electrodes were extracted from the cells at different states of charge (*i.e.* OCV, 0.01 V and 3 V). These were washed with dimethyl carbonate (DMC), dried under vacuum in the antechamber of an argon-filled glovebox and sealed under argon in a vial prior to measurement. Samples were analysed using a micro-focused monochromatic Al X-ray source (19.2 W) over an area of approximately 100 μm on a Thermo Fisher Scientific NEXSA spectrometer. Data were recorded at pass energies of 150 eV for survey scans and 40 eV for high-resolution scans with 1 eV and 0.1 eV step sizes respectively. Charge neutralisation of the sample was achieved using a combination of both low energy electrons and argon ions. To remove any surface contaminants, cluster cleaning was performed with 2 keV energy at 0.5 × 0.5 mm area for 60 s. Peak fitting and analysis was carried out using CASA-XPS software.

#### *Ex situ* solid-state nuclear magnetic resonance spectroscopy (NMR)

^19^F, ^7^Li, ^1^H and ^29^Si solid-state NMR spectra were obtained at 16.4 T on a Bruker Advance 700 MHz spectrometer (Bruker Biospin Corporation) operating at Larmor frequencies of 658.6, 272.1, 700.1 and 139.1 MHz, respectively. Spectra were referenced to PTFE (CF_2_ = −122 ppm), alanine (NH_3_ = 8.5 ppm) and kaolinite (−91.2 ppm) for ^19^F, ^1^H and ^29^Si, respectively. A 2D ^19^F–^7^Li heteronuclear correlation spectrum was obtained using a cross-polarisation based sequence with the contact pulse ramped for ^19^F. This spectrum was used as an internal reference for the ^7^Li spectra, (LiF = −1 ppm). Powdered samples were packed, in a nitrogen filled glovebox, into 2.5 mm MAS rotors, and rotated at MAS rates of 15–30 kHz. Pellet electrodes consisting of MXene powders, conductive carbon and PVDF binder in a 75 : 15 : 10 weight ratio were prepared by first mixing the components with a mortar and pestle and then adding small amounts of ethanol as the solvent. Around 25 mg of MXene powder were used in each pellet electrode. These electrodes were then cycled to different states-of-charge (*i.e.* discharged to 0.01 V and charged to 3 V) and then washed with DMC and dried under vacuum in the antechamber of a nitrogen filled glovebox. A pristine electrode was prepared to use as reference.

## Conflicts of interest

There are no conflicts to declare.

## Supplementary Material

NA-003-D1NA00081K-s001
