# Supplementary material for: Pillared Mo2TiC2 MXene for high-power and long-life lithium and sodium-ion batteries
Source: Nanoscale Adv. 2021 Apr 12;3(11):3145–58. doi: 10.1039/d1na00081k (PMC8168926; doi:10.1039/d1na00081k)
Supplement: NA-003-D1NA00081K-s001 [file NA-003-D1NA00081K-s001.pdf]

Electronic Supplementary Material (ESI) for Nanoscale Advances.
This journal is © The Royal Society of Chemistry 2021

Pillared Mo_2TiC_2 MXene for High-Power and Long-life Lithium and Sodium-ion Batteries

Philip A. Maughan[1], Luc Bouscarrat[1], Valerie R. Seymour[2], Shouqi Shao,[3] Sarah J. Haigh,[3] Richard Dawson[1], Nuria Tapia-Ruiz[2*], and Nuno Bimbo[1*#]

1 Department of Engineering, Lancaster University, Lancaster, LA1 4YW UK

2 Department of Chemistry, Lancaster University, Lancaster, LA1 4YB, UK

3 Department of Materials, University of Manchester, Manchester, M13 9PL UK

**Corresponding authors:*

Nuria Tapia-Ruiz, n.tapiaruiz@lancaster.ac.uk

Nuno Bimbo, n.bimbo@soton.ac.uk

[#]Current address: School of Chemistry, University of Southampton, Southampton, SO17 1BJ, UK

Table of Contents

Figure S1. STEM-EDS analysis of as-etched Mo_2TiC_2……Page 2
Figure S2. STEM-EDS spectrum of as-etched Mo_2TiC_2……Page 3
Figure S3. Raman spectra of the non-pillared, intercalated pillared and calcined pillared MXene materials……Page 4
Figure S4. HAADF-STEM and STEM-EDS analysis of Mo_2TiC_2-Si and Mo_2TiC_2-Si-400……Page 5
Figure S5. Pore size distribution for the pillared Mo_2TiC_2-Si-400 MXene……Page 6
Figure S6. Ti 2p XPS spectra for non-pillared and pillared Mo_2TiC_2……Page 7
Figure S7. Differential capacity plot (dQ dV^{-1}) for non-pillared Mo_2TiC_2……Page 8
Figure S8. Differential capacity plot (dQ dV^{-1}) for SiO_2-pillared Mo_2TiC_2……Page 9
Figure S9. Wide spectrum view of ex-situ ^7Li NMR of pillared MXene at 0.01 V……Page 10
Figure S10. Ex-situ ^1H NMR spectra of SiO_2-pillared Mo_2TiC_2……Page 11
Figure S11. Ex-situ Raman spectra of SiO_2-pillared Mo_2TiC_2……Page 12
Figure S12. Ex-situ ^7Li NMR spectra of SiO_2-pillared Mo_2TiC_2……Page 13
Figure S13. Ex-situ ^{19}F NMR spectra of SiO_2-pillared Mo_2TiC_2……Page 14
Figure S14. Cyclic voltammograms for non-pillared and pillared Mo_2TiC_2……Page 15
Figure S15. Cyclic voltammograms collected at increasing scan rates of 0.2, 0.5, 2 and 5 mV s^{-1} for non-pillared and pillared Mo_2TiC_2 and plots of b-value against voltage for non-pillared and pillared Mo_2TiC_2-Si-400……Page 16
Figure S16. Differential capacity plots (dQ dV^{-1}) for non-pillared and SiO_2-pillared Mo_2TiC_2 cycled vs. Na^+/Na……Page 17
Figure S17. Cyclic voltammograms collected at scan rates of 0.2, 0.5, 2 and 5 mV s^{-1} vs. Na^+/Na for non-pillared and pillared Mo_2TiC_2, and plots of *b*-values against voltage vs. Na^+/Na. for non-pillared and pillared-Mo_2TiC_2……Page 18
Figure S18. TGA analysis of as-etched Mo_2TiC_2……Page 19
Table S1. Comparison of interlayer spacings and surface areas of previously published pillared MXenes and porous Mo-based MXene materials……Page 20
Table S2. Comparison of the electrochemical performance of various Mo-based MXenes as negative electrodes in lithium-ion batteries……Page 21
Table S3. Comparison of the electrochemical performance of various Mo-based MXenes as negative electrodes in sodium-ion batteries……Page 22
Supporting Information References……Page 23

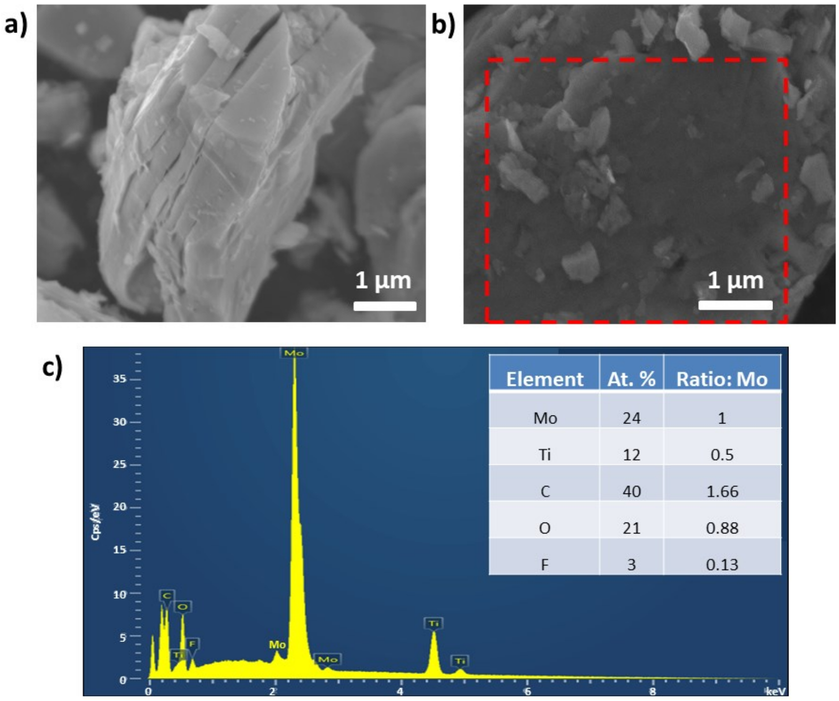

Figure S1. SEM-EDS analysis of as-etched Mo_2TiC_2. a-b) SEM micrographs of Mo_2TiC_2. The dashed red square in b) highlights the region analysed by EDS in c). c) EDS spectrum of the area highlighted in b), with quantification of the elements in the inset.

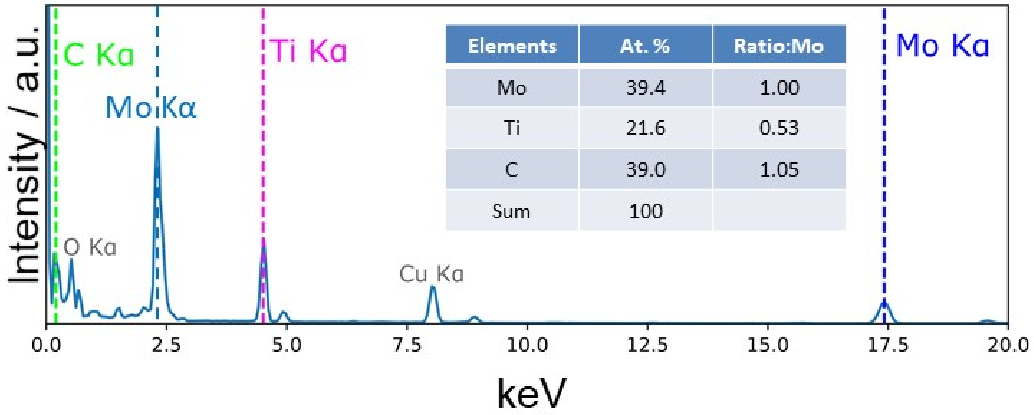

Elements	At. %	Ratio:Mo
Mo	39.4	1.00
Ti	21.6	0.53
C	39.0	1.05
Sum	100	

Figure S2. STEM-EDS spectrum of as-etched Mo_2TiC_2, with the inset showing the quantification of key MXene elements, Mo, Ti and C, from images in Figure 2 b-f in main manuscript.

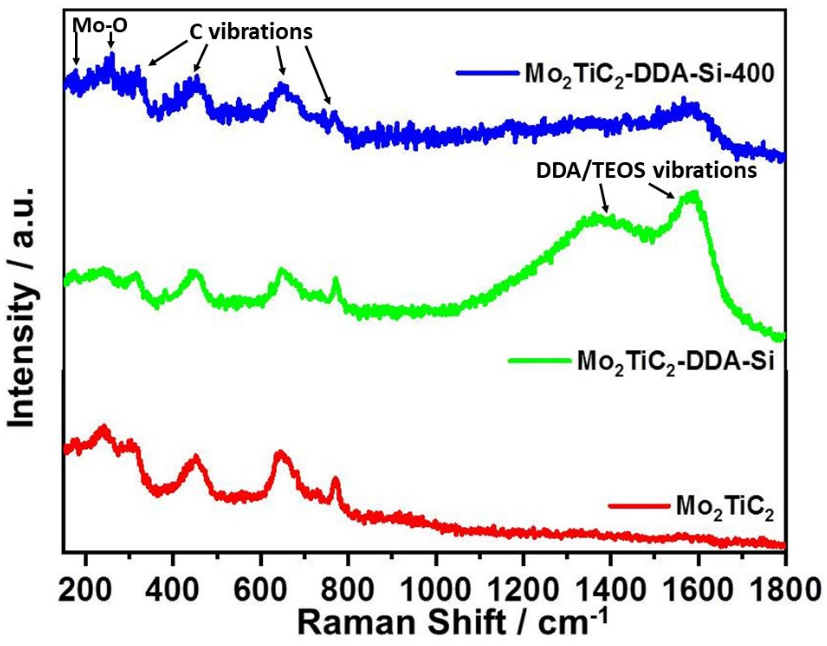

Figure S3. Raman spectra of the non-pillared (Mo_2TiC_2, red), pillared (Mo_2TiC_2-Si, green) and calcined (Mo_2TiC_2-Si-400, blue) MXene materials.

Raman modes can be observed at around 170, 240, 310, 450, 650 and 775 cm^{-1} in all samples, with no significant changes as a result of the pillaring process visible. These modes closely match previous reports on the Raman spectra of Mo_2TiC_2, giving further evidence to the successful synthesis of Mo_2TiC_2.[1,2] It has been reported that the peak around 170 cm^{-1} results from the *Eg* vibration of both Mo and Ti atoms in –O terminated Mo_2TiC_2.[2] The Raman mode at around 240 cm^{-1} corresponds directly to the *Eg* vibration of the O atoms.[2] This supports the EDS results, which suggested the presence of Mo-O in this MXene. The modes at 310, 450 and 650 and 750 cm^{-1} are all thought to mostly originate from the vibrations of C atoms in the MXene.[1,2] It should be noted that there are some differences in peak assignment in the literature, with Chen et al. assigning the 450 cm^{-1} peak to C vibrations, while Kim et al. assign it to -O surface vibrations.[1,2]

The intercalated pillared material shows additional Raman modes between 1000 and 1700 cm^{-1} which are much more intense than the MXene peaks. These broad peaks correspond to DDA vibrations, and match the modes seen in intercalated Ti_3C_2 Raman spectra in our previous work, suggesting that DDA has been successfully intercalated.[3] These modes are significantly reduced in the calcined material with only a small broad peak at 1600 cm^{-1} distinguishable, which shows that the majority of the carbon template has been removed after calcination, as was the case for the Ti_3C_2 MXene. However, a small amount of graphitic carbon remains in the structure, resulting in the small peak at 1600 cm^{-1}.

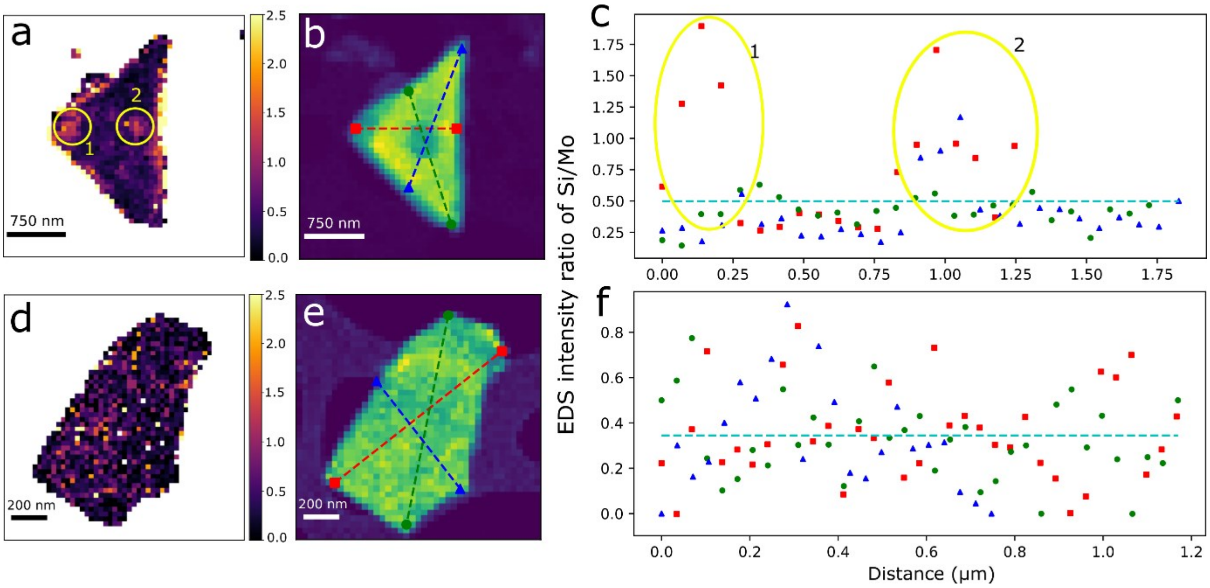

Figure S4. STEM-EDS maps showing the intensity ratio of Si to Mo, of (a) Mo_2TiC_2-Si and (d) Mo_2TiC_2-Si-400 flakes respectively, and their false coloured HAADF-STEM images shown in (b) and (e) where the red, green and blue dashed lines across the flake indicate the position of the line intensity scans shown in (c) and (f) respectively. The dashed lines indicate the mean values of the Si/Mo ratio. Two yellow circles labelled by 1 and 2 in (c) indicate Si-rich areas shown by the yellow circles in (a).

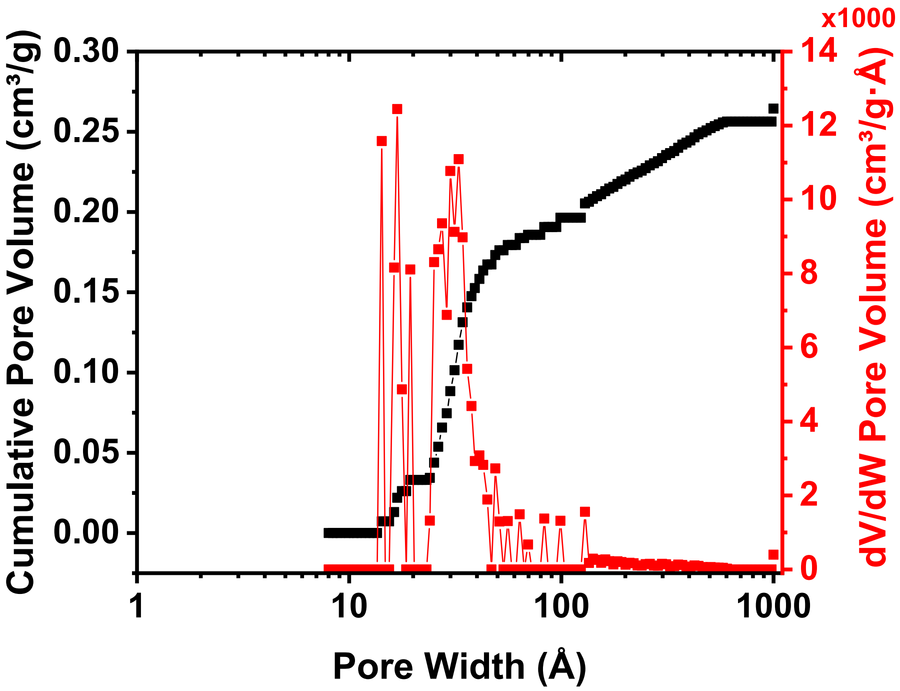

Figure S5. Pore size distribution for the pillared Mo_2TiC_2-Si-400 material, calculated using the non-linear density functional theory (NLDFT) method with a slit pore model from N_2 adsorption experiments carried out at 77 K.

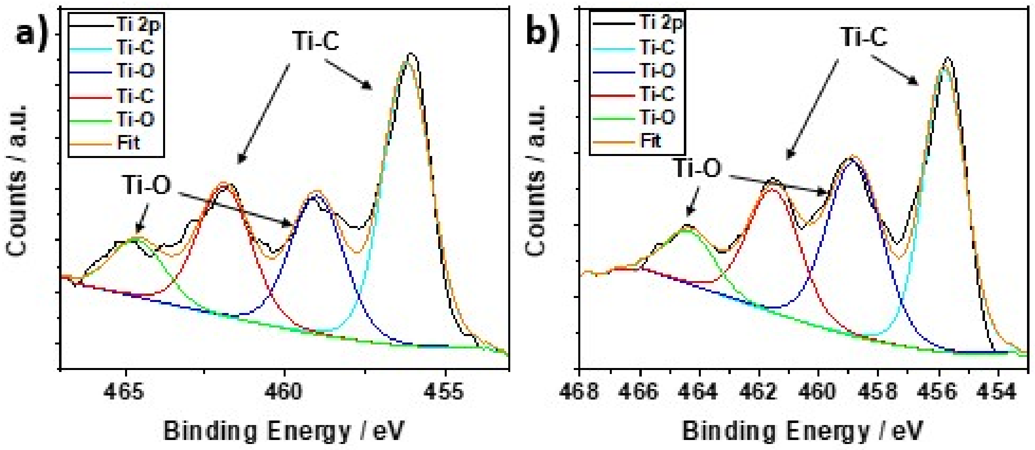

Figure S6. Ti 2p XPS spectra for a) non-pillared Mo_2TiC2_2 and b) SiO_2-pillared Mo_2TiC_2 after calcination at 400 °C, Mo_2TiC_2-Si-400.

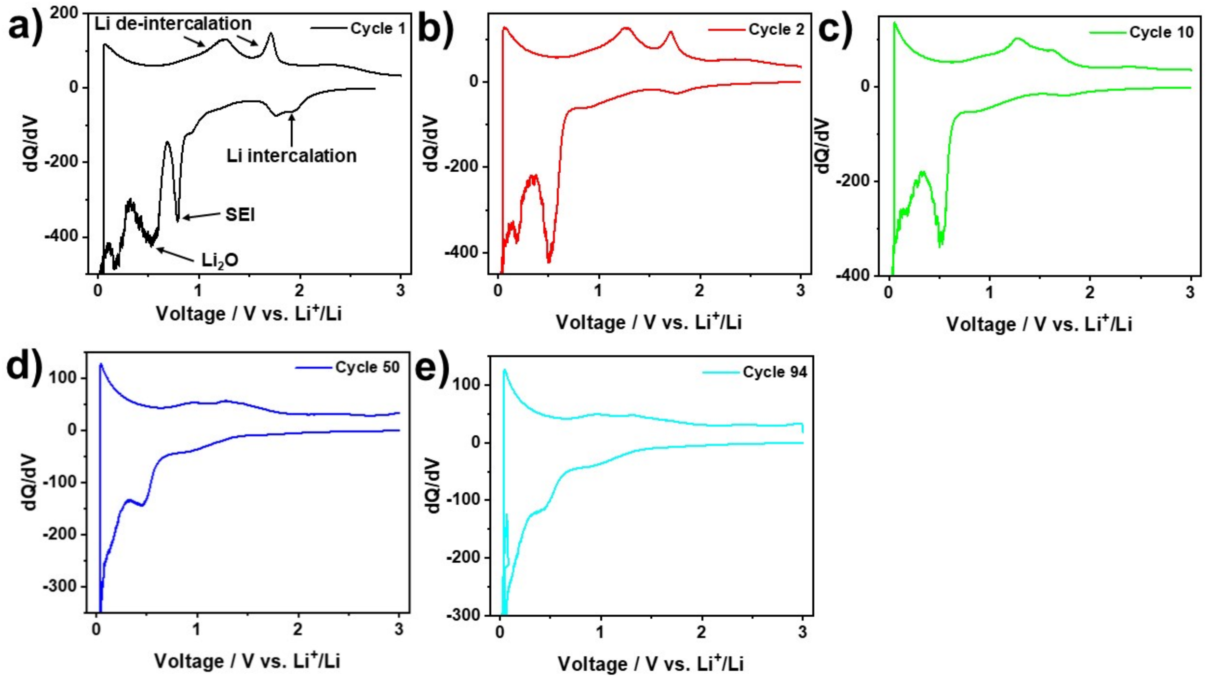

Figure S7. Differential capacity plots (dQ dV^{-1}) of selected cycles for non-pillared Mo_2TiC_2 cycled in the voltage range of 0.01-3 V vs. Li^+/Li at a current density of 20 mA g^{-1}. a) Cycle 1, b) cycle 2, c) cycle 10, d) cycle 50 and e) cycle 94.

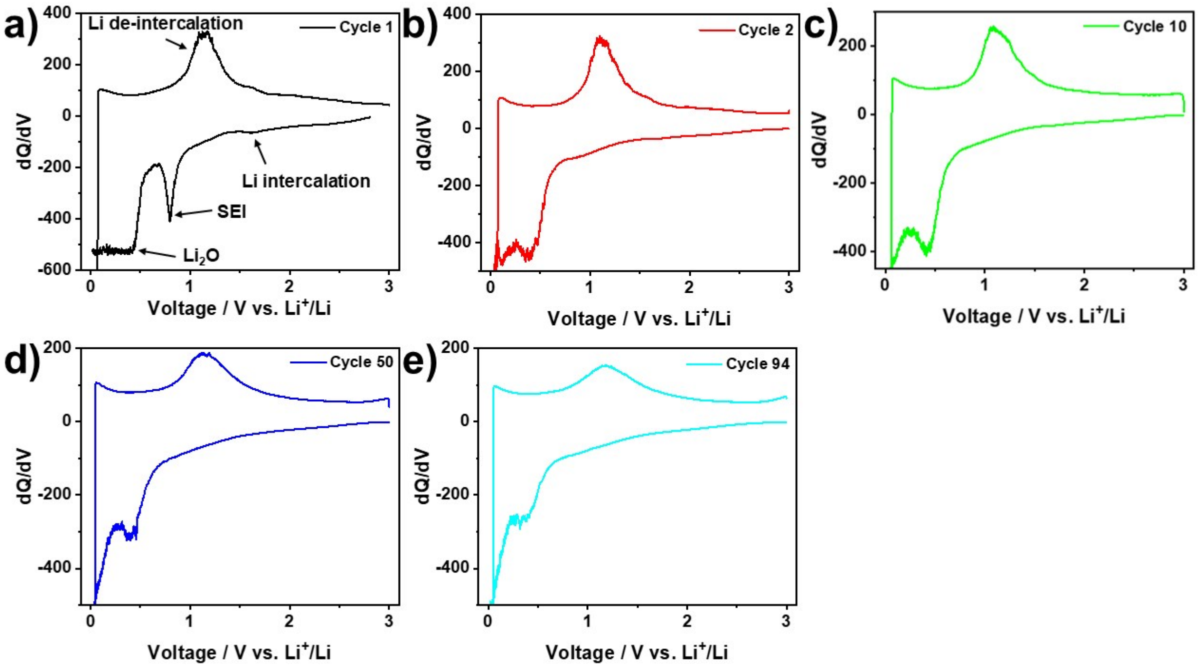

Figure S8. Differential capacity plots (dQ dV^{-1}) of selected cycles for SiO_2-pillared Mo_2TiC_2, Mo_2TiC_2-Si-400, cycled in the voltage range of 0.01-3 V vs. Li^+/Li at a current density of 20 mA g^{-1}. a) Cycle 1, b) cycle 2, c) cycle 10, d) cycle 50 and e) cycle 94.

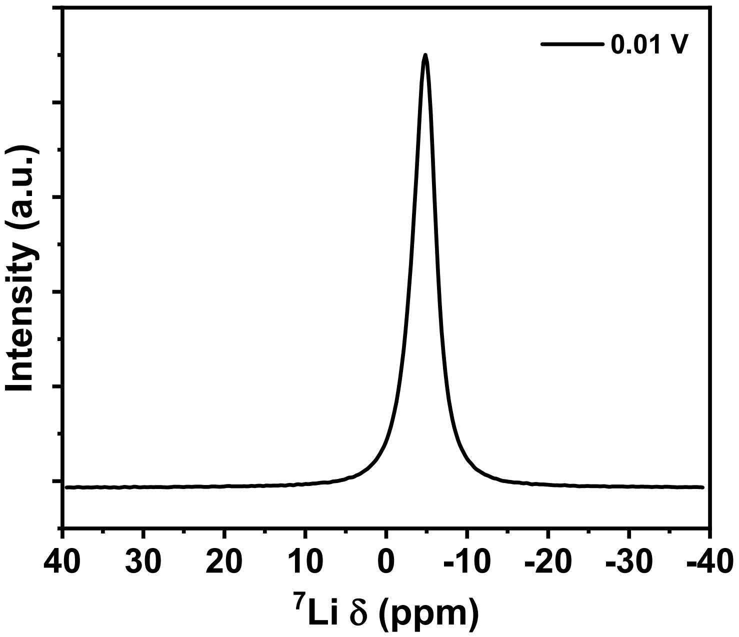

Figure S9. Wide-spectrum view of ex-situ ^{7}Li NMR of the 0.01 V discharged Mo_2TiC_2-Si-400 pillared MXene. There is no peak in the between 20 and 10 ppm, where Li_xSi_y alloys would be expected to form.[4]

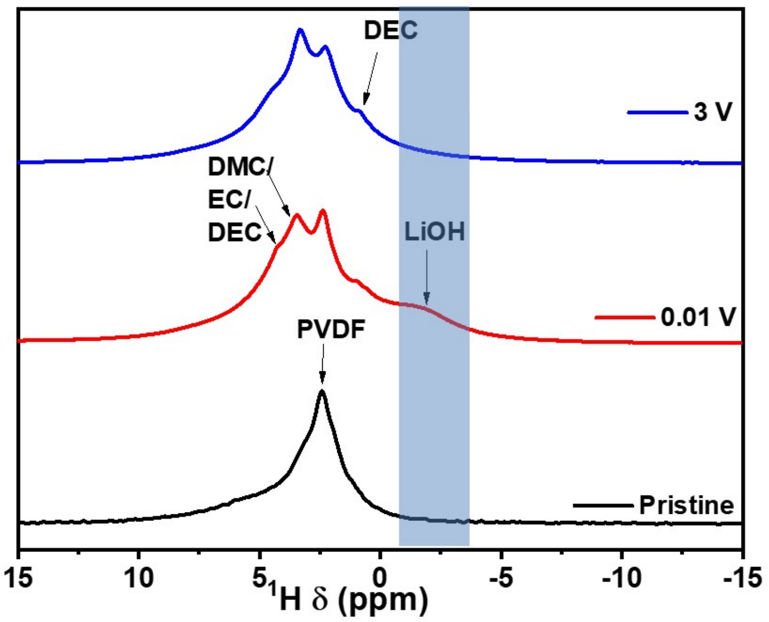

Figure S10. Ex-situ ^{1}H MAS NMR spectra (16.4 T, 30 kHz MAS) of Mo_2TiC_2-Si-400. Assignments are based on previous reports.[5]

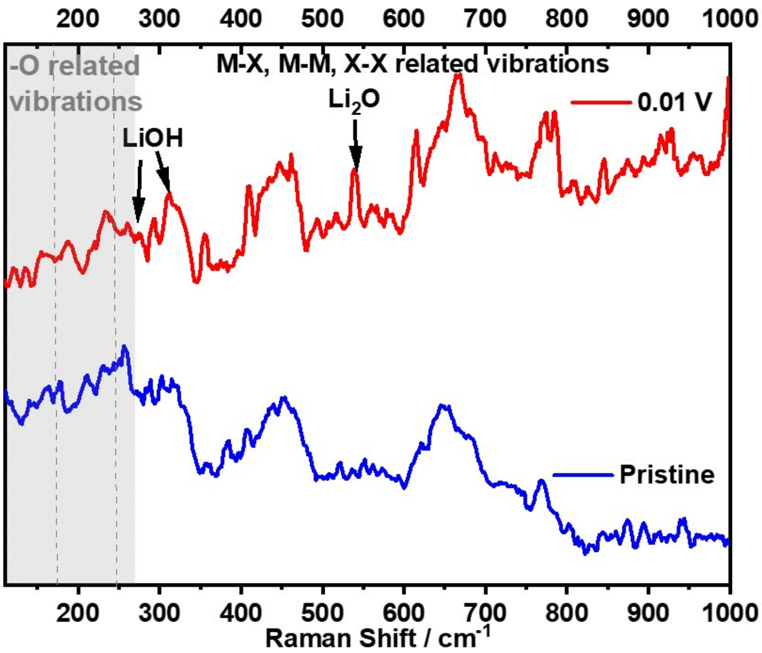

Figure S11. Ex-situ Raman spectra on the discharged pillared MXene, Mo_2TiC_2-Si-400. The pristine electrode is included for comparison. The grey area highlights the region where the vibrations correspond to the presence of Mo-O groups. The area with a white background shows the peaks which correspond to the main body of the MXene, M-M, M-X and X-X vibrations. A full description on the assignment of the MXene peaks can be found following Figure S3.

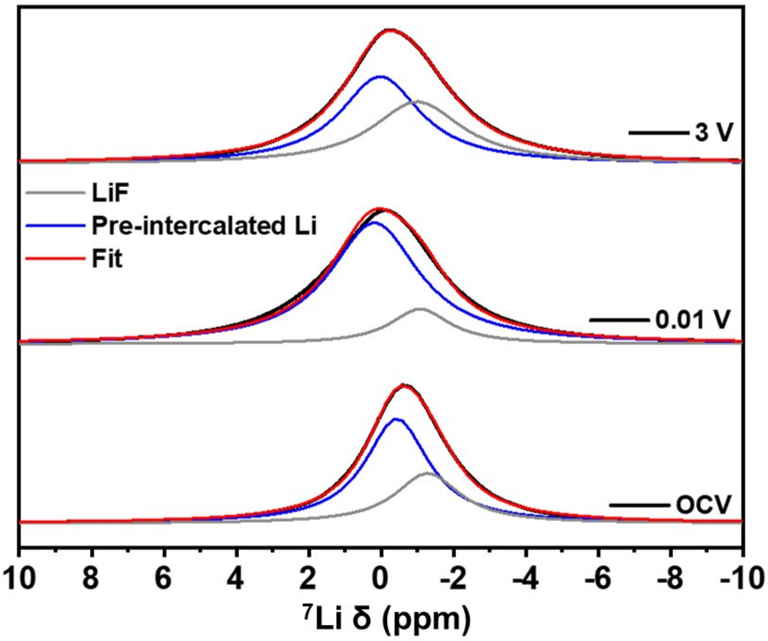

Figure S12. Ex-situ ^{7}Li MAS NMR spectra (16.4 T, 30 kHz MAS) of Mo_2TiC_2-Si-400. Assignments were based on previous reports on ^{7}Li NMR,[6,7] and our ^{19}F NMR results (Figure S13).

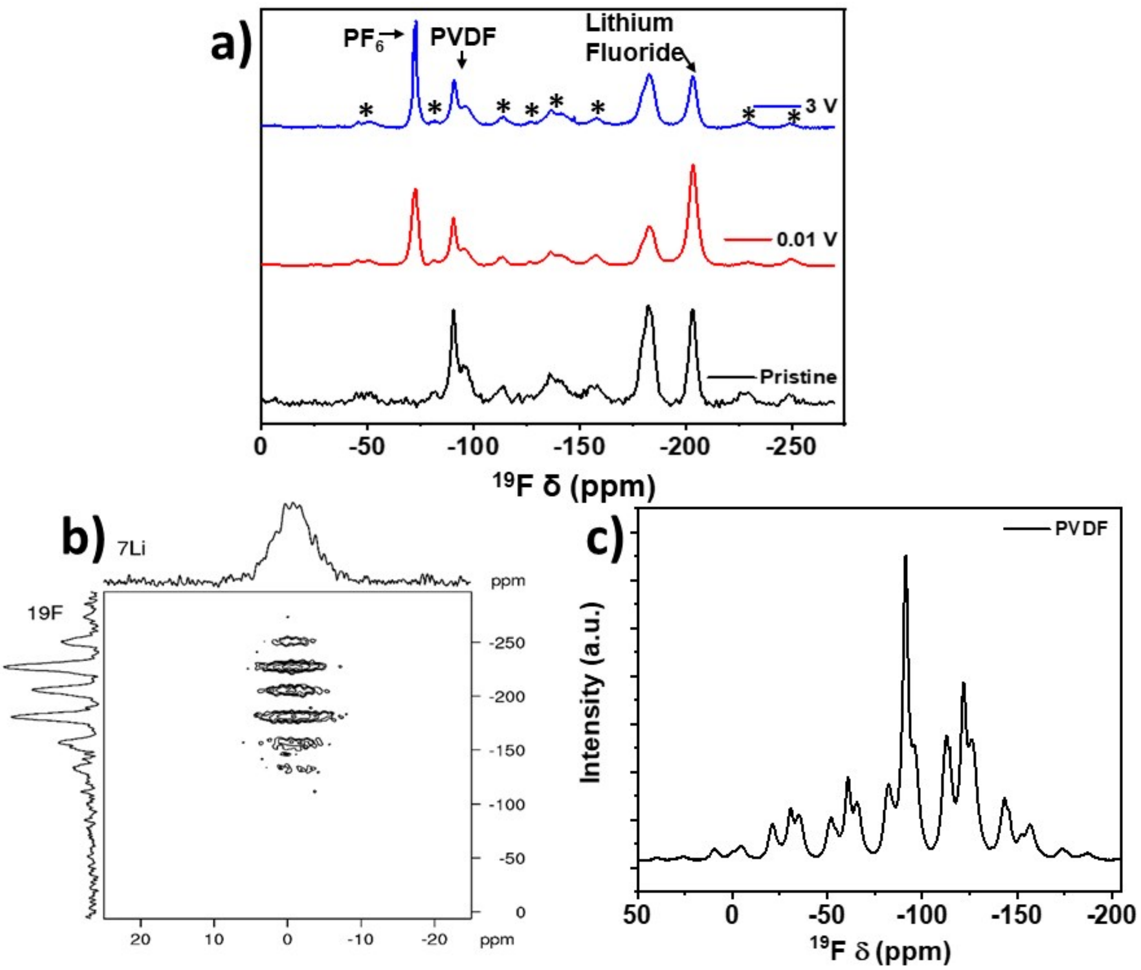

Figure S13. a) Ex-situ ^{19}F MAS NMR spectra (16.4 T, 30 kHz MAS) of SiO_2-pillared Mo_2TiC_2. * indicate spinning sidebands. Assignments are based on previous reports for fluorine compounds.[6] These are supported by: b) ^{19}F-^{7}Li HETCOR spectrum (Lithium fluoride) and c) ^{19}F MAS NMR spectrum of PVDF.

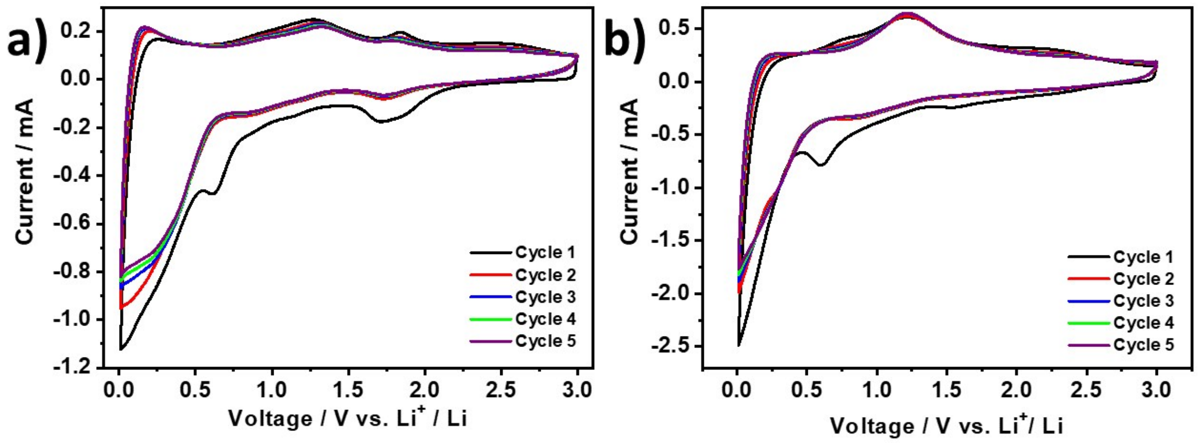

Figure S14. Cyclic voltammograms collected in the voltage range of 0.01-3 V vs. Li^+/Li at a scan rate of 0.2 mV s^{-1} for a) non-pillared Mo_2TiC_2 and b) pillared Mo_2TiC_2-Si-400.

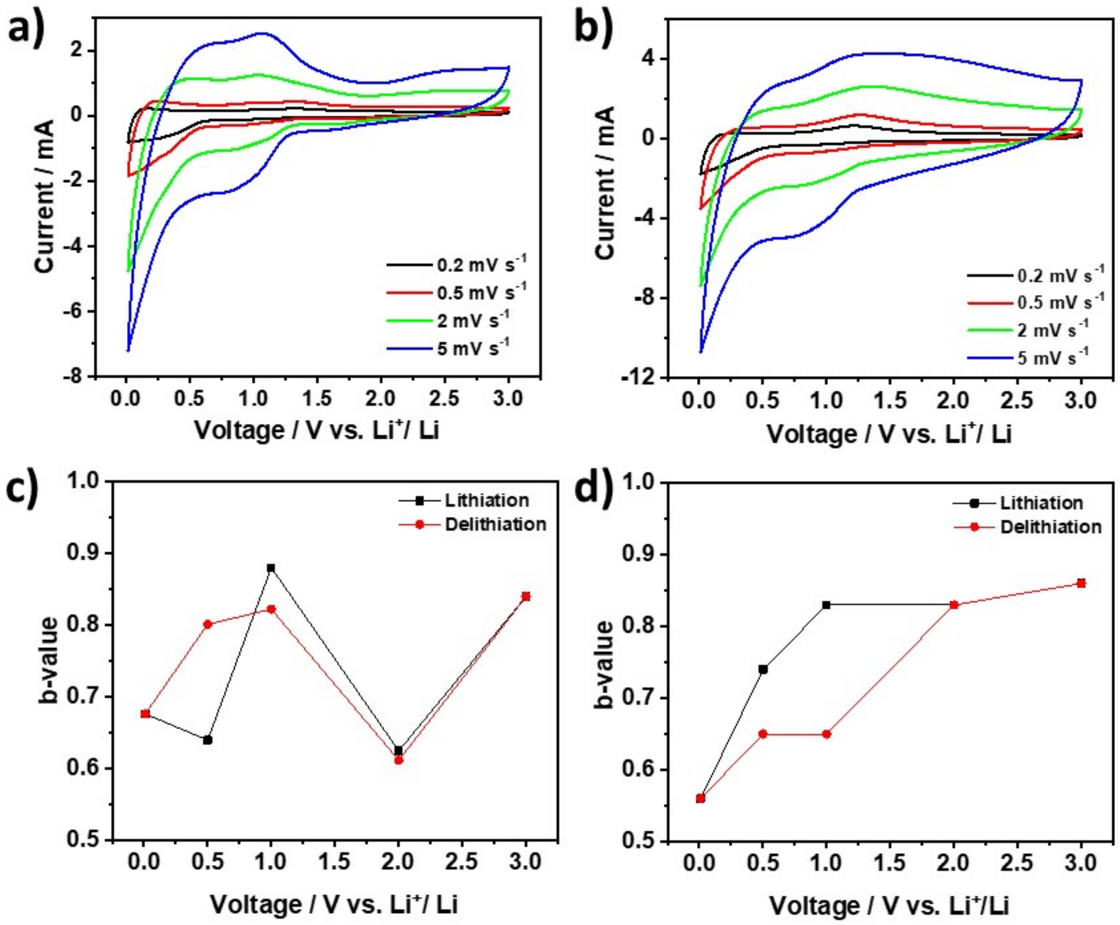

Figure S15. Cyclic voltammograms collected in the voltage range of 0.01-3 V vs. Li^+/Li at increasing scan rates of 0.2, 0.5, 2 and 5 mV s^{-1} for a) non-pillared Mo_2TiC_2 and b) pillared Mo_2TiC_2-Si-400. Plots of b-value against voltage for c) non-pillared Mo_2TiC_2 and d) pillared Mo_2TiC_2-Si-400.

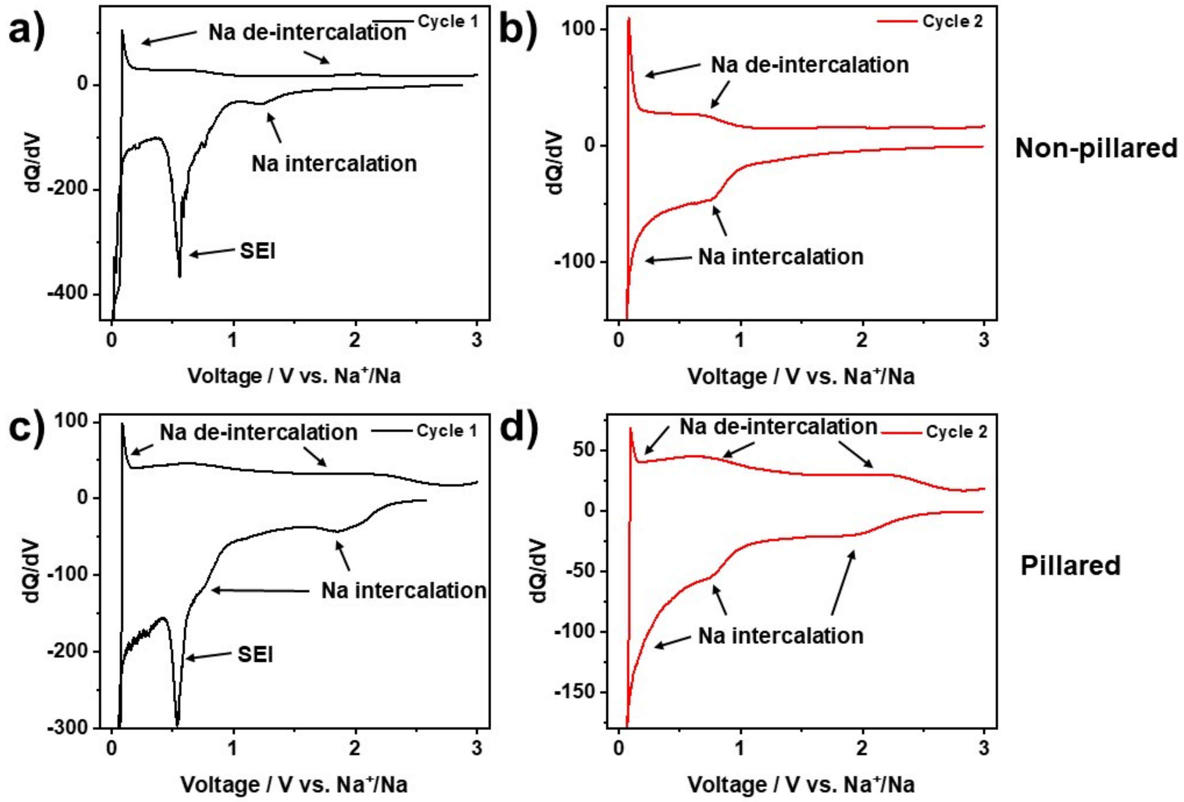

Figure S16. Differential capacity plots (dQ dV^{-1}) of selected cycles for non-pillared (top) and SiO_2-pillared (bottom) Mo_2TiC_2 cycled in a voltage range of 0.01-3 V vs. Na^+/Na at a current density of 20 mA g^{-1}. a) Cycle 1 non-pillared Mo_2TiC_2. b) Cycle 2 non-pillared Mo_2TiC_2. c) Cycle 1 pillared Mo_2TiC_2, Mo_2TiC_2-Si-400. d) Cycle 2 pillared Mo_2TiC_2, Mo_2TiC_2-Si-400.

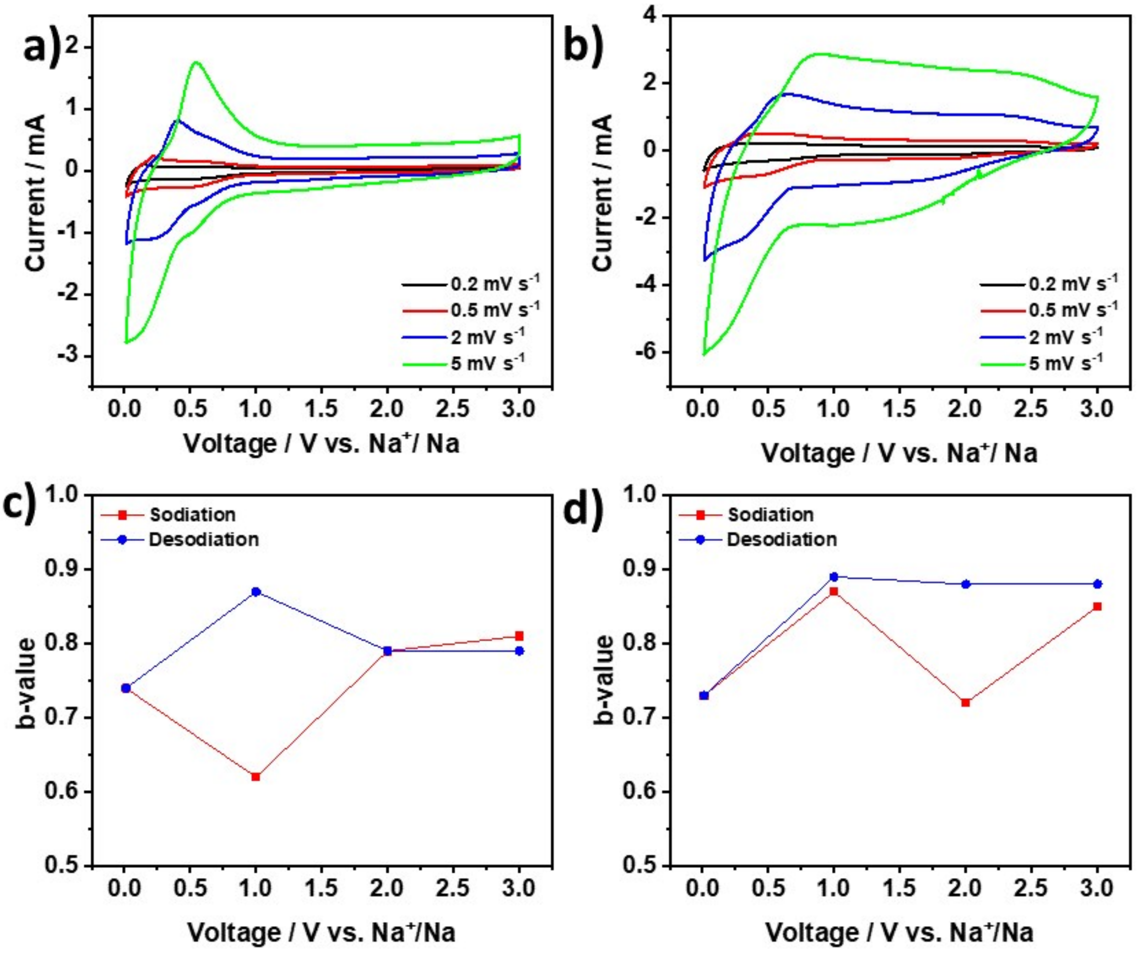

Figure S17. Cyclic voltammograms collected in the voltage range of 0.01-3 V vs. Na^{+}/Na at increasing scan rates of 0.2, 0.5, 2 and 5 mV s^{-1} for a) non-pillared Mo_2TiC_2 and b) pillared Mo_2TiC_2-Si-400. Plots of *b*-values against voltage vs. Na^{+}/Na. for c) non-pillared Mo_2TiC_2 and d) pillared-Mo_2TiC_2, Mo_2TiC_2-Si-400.

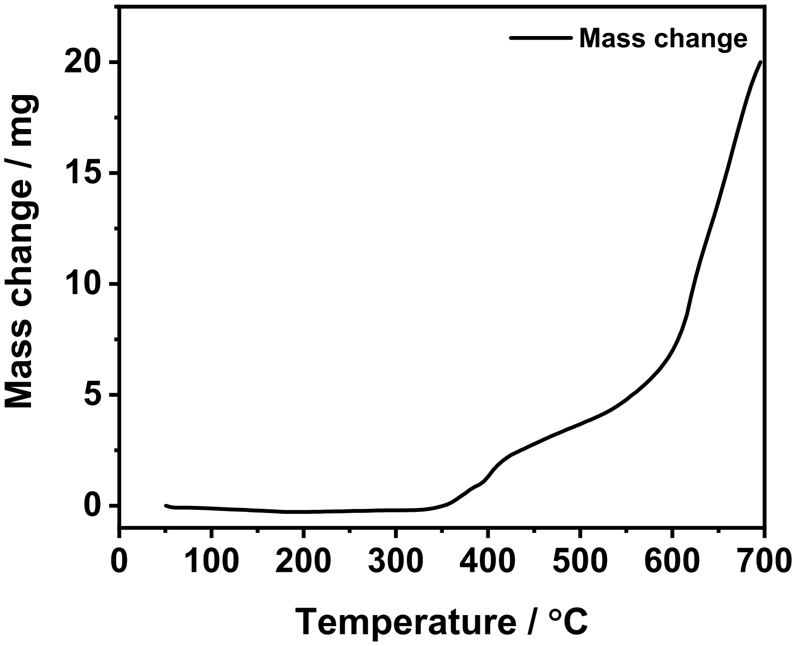

Figure S18. TGA plot showing the mass change of Mo_2TiC_2 against temperature. The thermal stability of Mo_2TiC_2 was investigated using TGA since this is directly relevant to the pillaring process, and has not been reported in the literature previously. Data were collected between 25 and 700 ˚C in air, with a heating rate of 1 ˚C min^{-1}. The mass changes very little up to around 350 ˚C, before increasing rapidly between 350-400 ˚C. This is followed by a period of more gradual mass increase between 400 and 600 ˚C with a very rapid increase between 600 and 700 ˚C. These results suggest that above 350 ˚C the MXene undergoes oxidation in a similar way to titanium based MXenes, likely resulting in the formation of Mo and Ti oxides. For the purpose of this study, the TGA results show that the material is susceptible to oxidation at temperatures exceeding 350 ˚C, so the calcination step used in the pillaring process (400 ˚C) was done under argon.

Table S1. Comparison of interlayer spacings and surface areas of previously published pillared MXenes and porous Mo-based MXene materials with the current work.

Material	Interlayer spacing (nm)	BET Surface area (m^2 g^{-1})	Reference
**CTAB and Sn^{4+} Pillared Ti_3C_2**	1.47-2.7	N/a	[8]
**Li, Na, K Pillared Ti_3C_2**	1.26	N/A	[9]
**CTAB and Sn^{2+} Pillared Ti_3C_2**	1.9-2.2	N/A	[10]
**TAEA Pillared Ti_3C_2**	1.38	N/A	[11]
**Pyrolised Amine Pillared Ti_3C_2**	0.99-2.83	9	[11]
**Ti_3C_2-CNT**	1.64	117	[12]
**SiO_2 Pillared Ti_3C_2**	1.49-4.24	235	[3]
**Mo_2C-CNT "thin film paper"**	1.9	N/A	[13]
**Mo_2C-TBAOH**	2.9	N/A	[13]
**Mesoporous Mo_2C (UV etched)**	1.75	N/A	[14]
**Mo_2C hydrothermally etched**	1.04	N/A	[15]
**TiO_2 Pillared MoS_2**	1.65	186 (Langmuir Surface area)	[16]
**$MoO_x(OH)_y$ Pillared MoS_2**	0.63	81	[14]
**Mo_2TiC_2 (HF etched)**	1.3	N/A	[17]
**Delaminated Mo_2TiC_2 (HF etched)**	2.1	N/A	[2]
**Mo_2TiC_2 (LiF etched)**	1.2	8	**This Work**
**SiO_2 Pillared Mo_2TiC_2 (LiF etched)**	2-3.6	202	**This Work**

Table S2. Comparison of the electrochemical performance of various Mo-based MXenes as negative electrodes in lithium-ion batteries, showing the superior performance of the SiO_2 pillared Mo_2TiC_2.

Material	Initial Capacity (mAh g^{-1})	2nd Cycle Capacity (mAh g^{-1})	Initial Coulombic Efficiency (%)	Capacity retention (%)	High Rate Capacity (mAh g^{-1})	Ref.
**Mo_2C-CNT "thin film paper" (HF etched)**	821 (10 mA g^{-1})	423 (10 mA g^{-1})	76%	132% (400 mA g^{-1}, 70 cycles)	~180 (5 A g^{-1}, ~ 138%, 1000 cycles	[13]
**Mesoporous Mo_2C (UV etched)**	~280 (5 mA g^{-1})	~170 (5 mA g^{-1})	~60%	~53% (5 mA g^{-1}, 140 cycles)	45 (1 A g^{-1})	[14]
**Mo_2TiC_2 (HF etched)**	311 (0.1 C)	~260 (0.1 C)	86%	92% (0.1 C, 25 cycles)	144 (1 C 82%, 160 cycles)	[17]
**Delaminated Mo_2TiC_2 (HF etched)**	268 (100 mA g^{-1})	134 (100 mA g^{-1})	50%	39% (100 mA g^{-1}, 100 cycles)	~0 (1 A g^{-1})	[2]
**Mo_2TiC_2 (LiF etched)**	344 (20 mA g^{-1})	219 (20 mA g^{-1})	64%	54% (20 mA g^{-1}, 94 cycles)	59 (1 A g^{-1})	**This Work**
**SiO_2 Pillared Mo_2TiC_2 (LiF etched)**	473 (20 mA g^{-1})	316 (20 mA g^{-1})	66%	80% (20 mA g^{-1}, 100 cycles)	143 (1 A g^{-1}, 80%, 500 cycles)	**This Work**

Table S3. Comparison of the electrochemical performance of various non Ti-based MXenes as negative electrodes in sodium-ion batteries.

Material	Initial Capacity (mAh g^{-1})	2nd Cycle Capacity (mAh g^{-1})	Initial Coulombic Efficiency (%)	Capacity retention (%)	High Rate Capacity (mAh g^{-1})	Ref.
**Mesoporous Mo_2C (UV etched)**	320 (5 mA g^{-1})	~70 (5 mA g^{-1})	15%	~70% (10 mA g^{-1}, 140 cycles)	~15 (1 A g^{-1})	[14]
**Mo_2TiC_2 (LiF etched)**	151 (20 mA g^{-1})	74 (20 mA g^{-1})	41%	65 (20 mA g^{-1}), 80 cycles	18 (1 A g^{-1})	**This Work**
**SiO_2 Pillared Mo_2TiC_2 (LiF etched)**	205 (20 mA g^{-1})	109 (20 mA g^{-1})	49%	75 (20 mA g^{-1}, 80 cycles)	40 (1 A g^{-1})	**This Work**

References

1. Kim, H., Anasori, B., Gogotsi, Y. & Alshareef, H. N. Thermoelectric Properties of Two-Dimensional Molybdenum-Based MXenes. *Chem. Mater.* **29**, 6472–6479 (2017).

2. Chen, C. *et al.* MoS_2 -on-MXene Heterostructures as Highly Reversible Anode Materials for Lithium-Ion Batteries. *Angew. Chemie Int. Ed.* **57**, 1846–1850 (2018).

3. Maughan, P. A. *et al.* Porous Silica-Pillared MXenes with Controllable Interlayer Distances for Long-Life Na-Ion Batteries. *Langmuir* (2020) doi:10.1021/acs.langmuir.0c00462.

4. Key, B. *et al.* Real-time NMR investigations of structural changes in silicon electrodes for lithium-ion batteries. *J. Am. Chem. Soc.* **131**, 9239–9249 (2009).

5. Hu, Y. Y. *et al.* Origin of additional capacities in metal oxide lithium-ion battery electrodes. *Nat. Mater.* **12**, 1130–1136 (2013).

6. Haber, S. & Leskes, M. What Can We Learn from Solid State NMR on the Electrode-Electrolyte Interface? *Adv. Mater.* **30**, 1706496 (2018).

7. Meyer, B. M., Leifer, N., Sakamoto, S., Greenbaum, S. G. & Grey, C. P. High field multinuclear NMR investigation of the SEI layer in lithium rechargeable batteries. *Electrochem. Solid-State Lett.* **8**, (2005).

8. Luo, J. *et al.* Pillared Structure Design of MXene with Ultralarge Interlayer Spacing for High-Performance Lithium-Ion Capacitors. *ACS Nano* **11**, 2459–2469 (2017).

9. Luo, J. *et al.* Tunable pseudocapacitance storage of MXene by cation pillaring for high performance sodium-ion capacitors. *J. Mater. Chem. A* **6**, 7794–7806 (2018).

10. Luo, J. *et al.* Pillared MXene with Ultralarge Interlayer Spacing as a Stable Matrix for High Performance Sodium Metal Anodes. *Adv. Funct. Mater.* **29**, 1805946 (2019).

11. Tian, W. *et al.* Layer-by-layer self-assembly of pillared two-dimensional multilayers. *Nat. Commun.* **10**, 1–10 (2019).

12. Xie, X. *et al.* Porous Heterostructured MXene/Carbon Nanotube Composite Paper with High Volumetric Capacity for Sodium-Based Energy Storage Devices. *Nano Energy* (2016) doi:10.1016/j.nanoen.2016.06.005.

13. Halim, J. *et al.* Synthesis and Characterization of 2D Molybdenum Carbide (MXene). *Adv. Funct. Mater.* **26**, 3118–3127 (2016).

14. Mei, J., Ayoko, G. A., Hu, C., Bell, J. M. & Sun, Z. Two-dimensional fluorine-free mesoporous Mo2C MXene via UV-induced selective etching of Mo2Ga2C for energy storage. *Sustain. Mater. Technol.* **25**, (2020).

15. Guo, Y. *et al.* Synthesis of two-dimensional carbide Mo2CTx MXene by hydrothermal etching with fluorides and its thermal stability. *Ceram. Int.* **46**, 19550–19556 (2020).

16. Paek, S.-M., Jung, H., Park, M., Lee, J.-K. & Choy, J.-H. An Inorganic Nanohybrid with High Specific Surface Area: TiO 2 -Pillared MoS 2. *Chem. Mater.* **17**, 3492–3498 (2005).

17. Anasori, B. *et al.* Two-Dimensional, Ordered, Double Transition Metals Carbides (MXenes). *ACS Nano* **9**, 9507–16 (2015).
